# Synaptic Dysfunction in the Anterior Cingulate Cortex Underlies Pain‐Anxiety Comorbidity in a Mandibular Asymmetry Mouse Model

**DOI:** 10.1002/advs.202509509

**Published:** 2025-09-24

**Authors:** Zhaoyichun Zhang, Yanran Zhang, Jialin Si, Honghui Mao, Feng He, Jin Ning, Guaiguai Ma, Xiaohua Chen, Haoxiang Xiao, Yuanyuan Zhu, Haifeng Zhang, Yifan Lu, Qian Liu, Meng Nian, Shiquan Sun, Shibin Yu, Shengxi Wu, Ze Fan, Zuolin Jin, Jing Huang

**Affiliations:** ^1^ State Key Laboratory of Oral & Maxillofacial Reconstruction and Regeneration National Clinical Research Center for Oral Diseases Shaanxi Clinical Research Center for Oral Diseases Department of Orthondotics School of Stomatology The Fourth Military Medical University Xi'an 710032 China; ^2^ Department of Neurobiology Basic Medical Science Academy The Fourth Military Medical University Xi'an 710032 China; ^3^ State Key Laboratory of Oral & Maxillofacial Reconstruction and Regeneration National Clinical Research Center for Oral Diseases Shaanxi Clinical Research Center for Oral Diseases Department of Pediatric Dentistry School of Stomatology The Fourth Military Medical University Xi'an 710032 China; ^4^ State Key Laboratory of Oral & Maxillofacial Reconstruction and Regeneration National Clinical Research Center for Oral Diseases Shaanxi International Joint Research Center for Oral Diseases Department of Oral Anatomy and Physiology School of Stomatology The Fourth Military Medical University Xi'an 710032 China; ^5^ Center for Single‐Cell Omics and Health School of Public Health Xi'an Jiaotong University Xi'an 710061 China; ^6^ State Key Laboratory of Oral and Maxillofacial Reconstruction and Regeneration National Clinical Research Center for Oral Diseases Shaanxi Engineering Research Center for Dental Materials and Advanced Manufacture Department of Anesthesiology School of Stomatology The Fourth Military Medical University Xi'an 710032 China

**Keywords:** anterior cingulate cortex, anxiety, mandibular asymmetry, multi‐omics analysis, pain

## Abstract

Structural craniofacial abnormalities, particularly mandibular asymmetry (MA), are increasingly recognized as key drivers of orofacial pain and emotional comorbidities, although the underlying neural mechanisms remain unclear. This study aims to develop a preclinical MA model to investigate the dynamic interplay between craniofacial structural defects and neurobehavioral dysfunction. Longitudinal behavioral phenotyping including von Frey filaments test as well as open field and elevated plus maze tests reveals progressive sensory hypersensitivity and anxiety‐like behaviors, with computational ethology revealing subtle but consistent alterations in the naturalistic behaviors. Whole‐brain activity mapping reveal hyperactivation in the anterior cingulate cortex (ACC), whereas multi‐omics profiling reveal cell type‐specific transcriptional changes and synaptic reorganization within this region. Functional investigation including sparse labeling, electrophysiology, western blotting, and chemogenetics demonstrate that the ACC modulation regulated both pain and anxiety. These findings establish a causal association between structural craniofacial defects and maladaptive neural circuit remodeling, with the ACC emerging as a critical therapeutic target. The study provides a comprehensive framework for understanding how anatomical abnormalities translate into persistent neurological dysfunction and offers new avenues for mechanistically informed intervention.

## Graphical abstract

This study establishes a mandibular asymmetry (MA) mouse model that recapitulates chronic orofacial pain and anxiety comorbidity. Anterior cingulate cortex (ACC) hyperactivation and synaptic dysfunction are identified as central neural mechanisms underlying these pathologies, with chemogenetic inhibition of ACC CaMKII+ neurons effectively reversing both sensory and affective phenotypes, revealing novel targets for neuromodulation‐based therapeutic intervention.

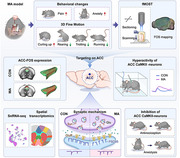

## 1 Introduction

Temporomandibular disorders (TMDs), characterized by jaw joint pain, clicking or popping sounds, and restricted mandibular movement, often progress to chronic pain and multisystem functional impairments, including headaches, neck pain, and emotional disturbances, such as anxiety and depression.^[^ ^1^, ^2^ ^]^ Although the sensory and emotional comorbidities of TMDs significantly reduce the quality of life, the neurobiological mechanisms underlying these dysfunctions remain poorly understood, hindering the development of targeted therapies. Craniofacial structural abnormalities, particularly dentofacial or MA, may represent a critical underlying etiological factor in TMDs.^[^ ^3^, ^4^ ^]^ MA frequently coexists with TMD‐related pathologies, including abnormal condylar morphology,^[^ ^5^ ^]^ articular disc displacement,^[^ ^6^ ^]^ and chronic temporomandibular joint (TMJ) inflammation.^[^ ^7^ ^]^ These structural deviations induce persistent nociceptive signaling through trigeminal nerve activation, which may drive central sensitization and neuroplastic remodeling of pain‐processing circuits.^[^ ^8^, ^9^ ^]^ Critically, chronic pain from MA engages in a bidirectional interplay with emotional regulation systems; prolonged nociceptive input alters functional connectivity in the limbic and cortical regions, whereas stress‐induced sympathetic hyperactivity exacerbates masticatory muscle tension and pain perception, creating a self‐perpetuating cycle of physical and psychological dysfunction.^[^ ^10^, ^11^ ^]^ This vicious cycle highlights the urgency of dissecting the neural mechanisms bridging structural craniofacial defects to pain–emotion comorbidities.

With the increasing number of studies on the orofacial–brain axis, the role of the central nervous system in orofacial diseases has become of utmost importance.^[^ ^12^, ^13^ ^]^ In patients with TMD, joint injury or inflammation activates nociceptors, generating pain signals that are transmitted to the central nervous system via the trigeminal nerve.^[^ ^14^, ^15^ ^]^ Long‐term peripheral nociceptive input induces central sensitization, enhances responsiveness of pain‐processing circuits, and lowers pain thresholds,^[^ ^16^ ^]^ facilitating the transition from acute to chronic pain.^[^ ^17^ ^]^ Concurrently, chronic pain drives structural and functional remodeling of the brain,^[^ ^18^, ^19^ ^]^ including aberrant connectivity in the affective and somatosensory networks. For example, functional magnetic resonance imaging studies in patients with TMDs have revealed hyperactivity and disrupted communication between the ACC (anterior cingulate cortex, also referred to as ACA in the Allen Reference Atlas), dorsolateral prefrontal cortex, and insular regions, which are critical for pain–emotion integration and cognitive control.^[^ ^20^ ^]^ This dysregulation manifests clinically as heightened pain perception, anxiety, and depression, which, in turn, exacerbate TMD pathophysiology. Negative emotions activate sympathetic pathways, thereby increasing masticatory muscle tension^[^ ^21^ ^]^ and pain sensitivity,^[^ ^22^ ^]^ whereas pain further amplifies emotional distress and creates a self‐reinforcing feedback loop. Despite these advances, the role of the ACC as a hub for TMD‐related pain–emotion comorbidities remains underexplored, particularly in the context of structural craniofacial abnormalities, such as MA. Furthermore, no preclinical studies have linked MA to longitudinal pain–emotion dynamics and brain‐wide circuit alterations, leaving critical gaps in our understanding of how and when these pathologies arise.

In the present study, we aimed to establish a mouse model of MA to systematically investigate TMD‐related sensory and emotional pathophysiology. Longitudinal behavioral profiling at multiple time points revealed distinct phases of facial mechanical allodynia and anxiety‐like behaviors. At the peak symptom stage, deep learning‐based quantitative analysis of spontaneous behaviors generated high‐resolution ethological profiles of the MA‐induced abnormalities. Whole‐brain mapping using fluorescence micro‐optical sectioning tomography (fMOST) and FOS tracing identified the ACC as a key hub for MA‐induced neuronal activation. Subsequent single‐cell and spatial transcriptomic analyses of the ACC revealed molecular and cellular reprogramming events, and ultrastructural and electrophysiological studies demonstrated MA‐driven synaptic plasticity alterations. Crucially, chemogenetic modulation of ACC activity ameliorated both the pain and anxiety phenotypes, validating its functional centrality. By integrating multi‐omics, circuit‐level, and behavioral approaches, this study not only delineates ACC‐dependent mechanisms linking dentofacial structural abnormalities to chronic pain–emotion comorbidity but also establishes a translational roadmap for precision neuromodulation in TMD.

## 2 Results

### 2.1 Pathological and Behavioral Characterization of the Mandibular Asymmetry Mouse Model

**Figure 1:**
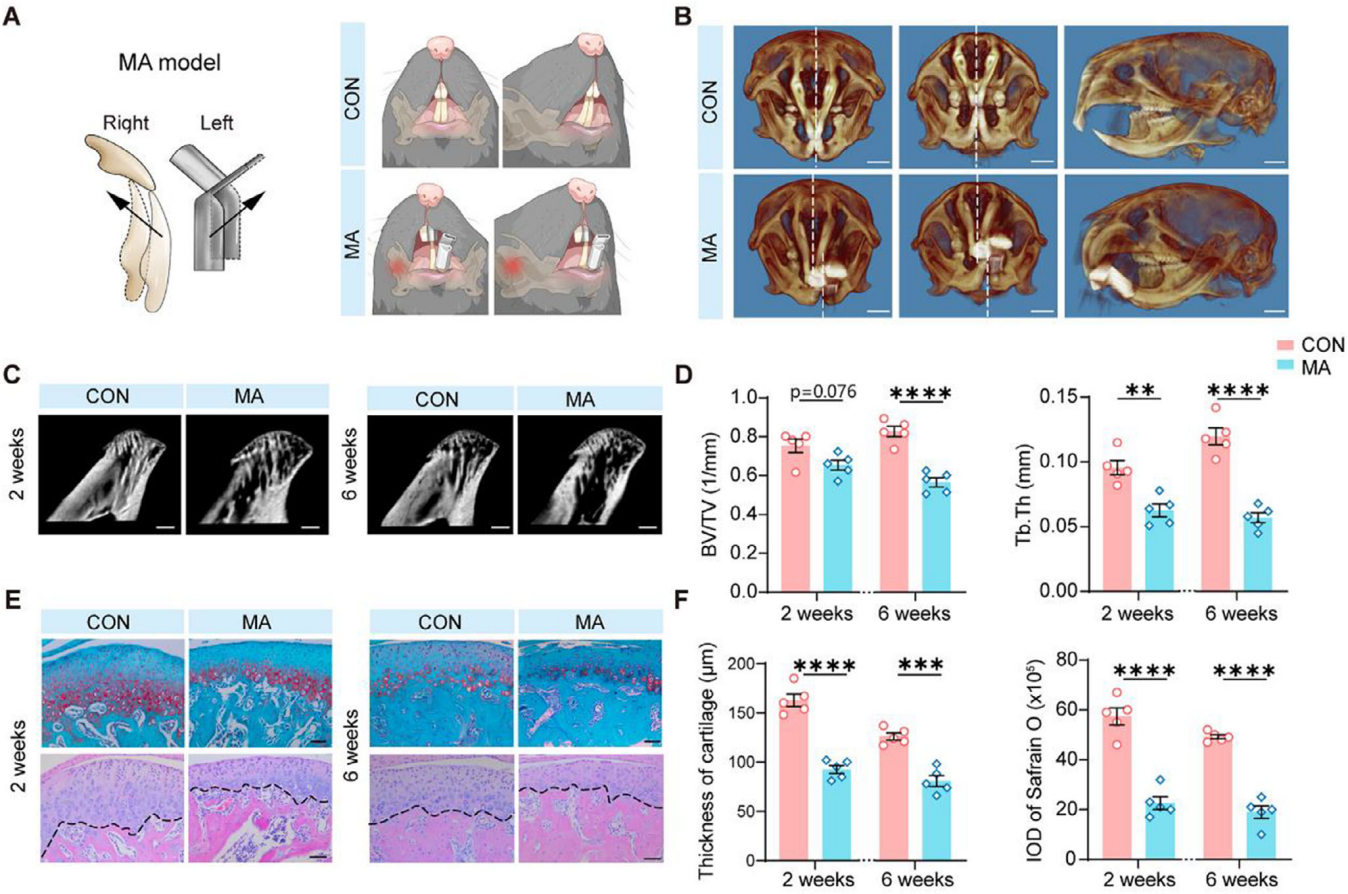
Establishment and pathological features of the MA model. A) Schematic illustration of the method for establishing the MA model by placing a custom‐designed oral device to disrupt normal dental occlusion and create a mandibular asymmetry. B) CT 3D reconstruction images, showing that two weeks after model establishment, the MA group exhibits severe mandibular asymmetry compared with the CON group. Scale bars: 2000 µm. C) Micro‐CT images of trabecular bones in MA and CON group mice at two and six weeks after model establishment. Scale bars: 500 µm. D) Quantitative analysis of bone volume (BV) fraction and trabecular bone thickness (TB) (n = 5). E) Safranin O staining and hematoxylin‐eosin (HE) staining of the CON and MA groups at 2‐ and 6‐week time points. Scale bars: 50 µm. F) Bar graphs showing the thickness of cartilage and the integrated optical density (IOD) of safranin O staining in the CON and MA groups at 2‐ and 6‐week time points (n = 5). ^**^P < 0.01, ^***^P < 0.001, ^****^P < 0.0001, by Two‐tailed unpaired t test, Mann‐Whitney U test. Data are presented as the mean ± SEM.

To develop a new animal model capable of accurately mimicking MA‐related pathological alterations and behavioral deficits observed clinically, we placed a custom‐designed oral device in the mouth of a mouse to disrupt normal dental occlusion, thereby creating a condition of unilateral posterior crossbite (**Figure** **1**A). Two weeks after model establishment, using the computed tomography (CT) 3D reconstruction technique, we found that the MA group showed severe mandibular asymmetrical symptoms compared with the control (CON) group (Figure 1B). Meanwhile, at both 2 and 6 weeks after model establishment, alterations in the trabecular bones of the MA mice were observed compared with those of the CON group mice (Figure 1C, Video S1, Supporting Information). Specifically, this manifested as a decrease in the bone volume (BV) fraction and thickness of the trabecular bones (TB.Th) (Figure 1D). To verify the pathological changes in the MA model, we used safranin O and hematoxylin–eosin staining, which demonstrated that MA led to a reduction in the number of chondral granule cells and a decrease in the thickness of the cartilage layer (Figure 1E,F). Consistent with the structural and pathological changes, the International Osteoarthritis Research Society scores were significantly higher in the MA group than in the CON group.

**Figure 2:**
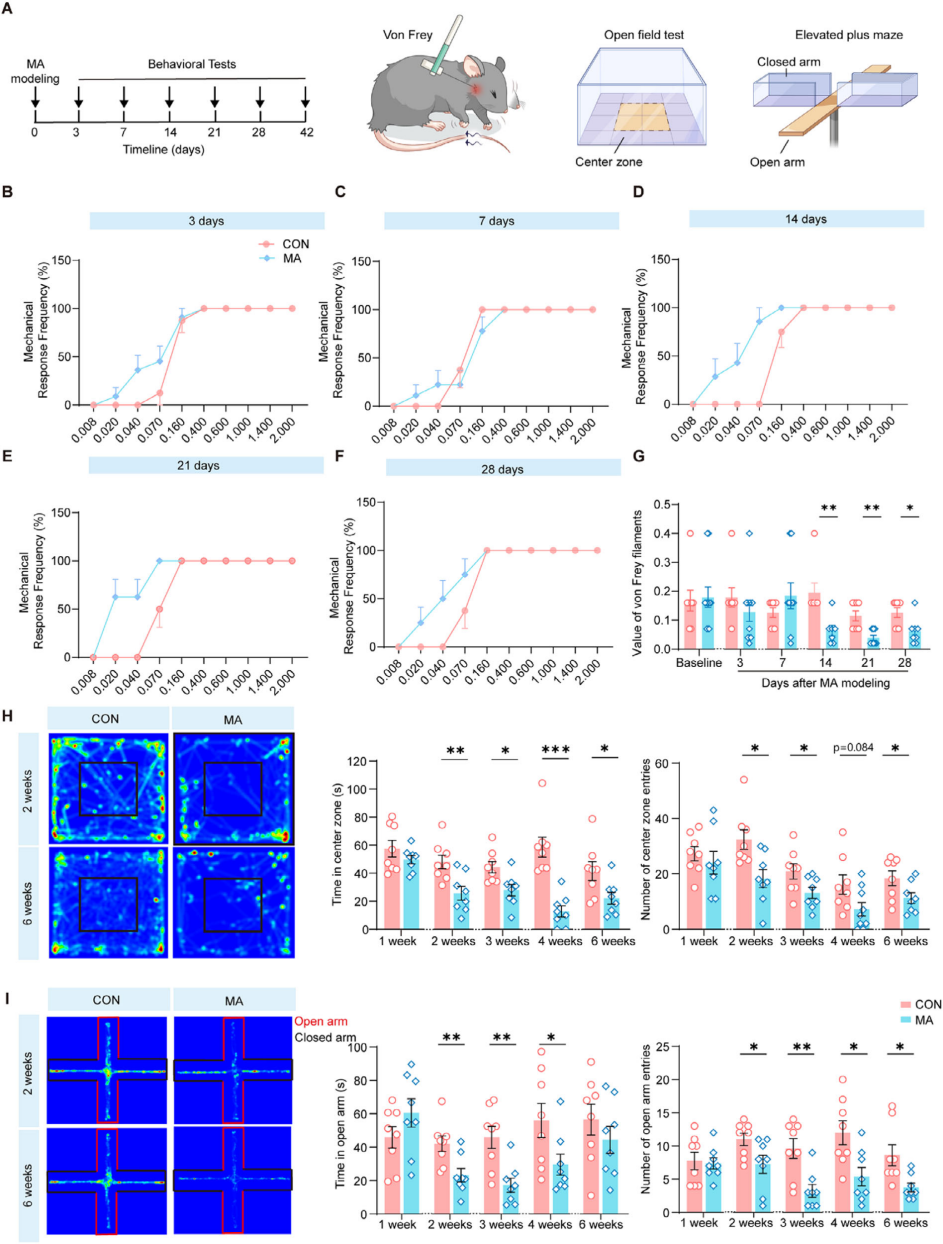
Pain and anxiety‐related behaviors in male MA model mice. A) A timeline diagram illustrating the experimental schedule. B–F) The von Frey test assesses pain sensitivity by measuring the mechanical response frequency as a percentage. Sub‐figures B–F show data at different time points (3, 7, 14, 21, and 28 days) after MA modeling. The plots display the mechanical response frequencies of the CON (pink) and MA (blue) groups to different von Frey filament forces (n = 7–11). G) A graph presenting the "pain value" of von Frey filaments at various time points after MA modeling. The baseline data is contrasted with data at days 3, 7, 14, 21, and 28 (n = 7–11). H) Results of the open field test. Heatmaps (left) display the movement patterns of mice in the open field for the CON and MA groups at 2 and 6 weeks. Bar graphs (right) quantify the time spent in the center zone and the number of center zone entries for both groups over different time periods (1 week to 6 weeks) (n = 8). I) Results of the elevated plus maze test. Heatmaps (left) show visual representations of the movement of mice in the open and closed arms of the maze for the CON and MA groups at 2 and 6 weeks. Bar graphs (right) quantify the time spent in the open arm and the number of open arm entries for both groups over different time periods (1–6 weeks) (n = 8). ^*^P < 0.05, ^**^P < 0.01, ^***^P < 0.001, ^****^P < 0.0001, by Two‐tailed unpaired separate variance estimation t‐test, Two‐tailed unpaired t test and Mann‐Whitney U test, Friedman M test. Data are presented as the mean ± SEM.

To systematically characterize MA‐induced behavioral alterations, we first used the von Frey test to evaluate the pain sensitivity of male mice and found that the MA model led to persistent mechanical hyperalgesia, reflecting a chronic pain behavioral phenotype (**Figure** **2**A–F). Notably, on the 14th day after model establishment, the MA group exhibited a significantly lower mechanical pain threshold compared with the CON group, and this hypersensitivity persisted through day 28 (Figure 2G). Previous research has indicated that chronic pain can elicit modifications in emotional behaviors.^[^ ^23^ ^]^ Therefore, we performed an open field test and an elevated plus‐maze experiment to assess the anxiety level of the male MA group. The results of the behavioral experiments demonstrated that, in the open field test, compared with the CON group, the MA group spent less time in the central area and had fewer entries into it (Figure 2H), with a reduction in the total distances and the distances in the central zone (Figure S1A, Supporting Information). Furthermore, in the elevated plus‐maze test, compared with the CON group, the MA group spent less time, had fewer entries, and covered shorter distances in the open arms (Figure 2I), whereas there was no significant difference in total distances (Figure S1B, Supporting Information). These behavioral results indicate that the MA group exhibited markedly elevated anxiety‐like phenotypes compared to the CON group. To further investigate whether the pain‐anxiety comorbidity in the MA model is gender‐related, we conducted the same behavioral experiments in female mice and found that, compared with the CON group, the pain threshold in the female MA mice was also significantly decreased (Figure S2A‐B, Supporting Information). Similar to their male counterparts, the female MA mice also exhibited anxiety‐like behaviors in the open field and elevated plus maze tests (Figure S2C‐F, Supporting Information). These behavioral experiments indicate that the ACC‐mediated pain‐anxiety comorbidity in the MA model operates through fundamentally similar pathways regardless of sex.

**Figure 3:**
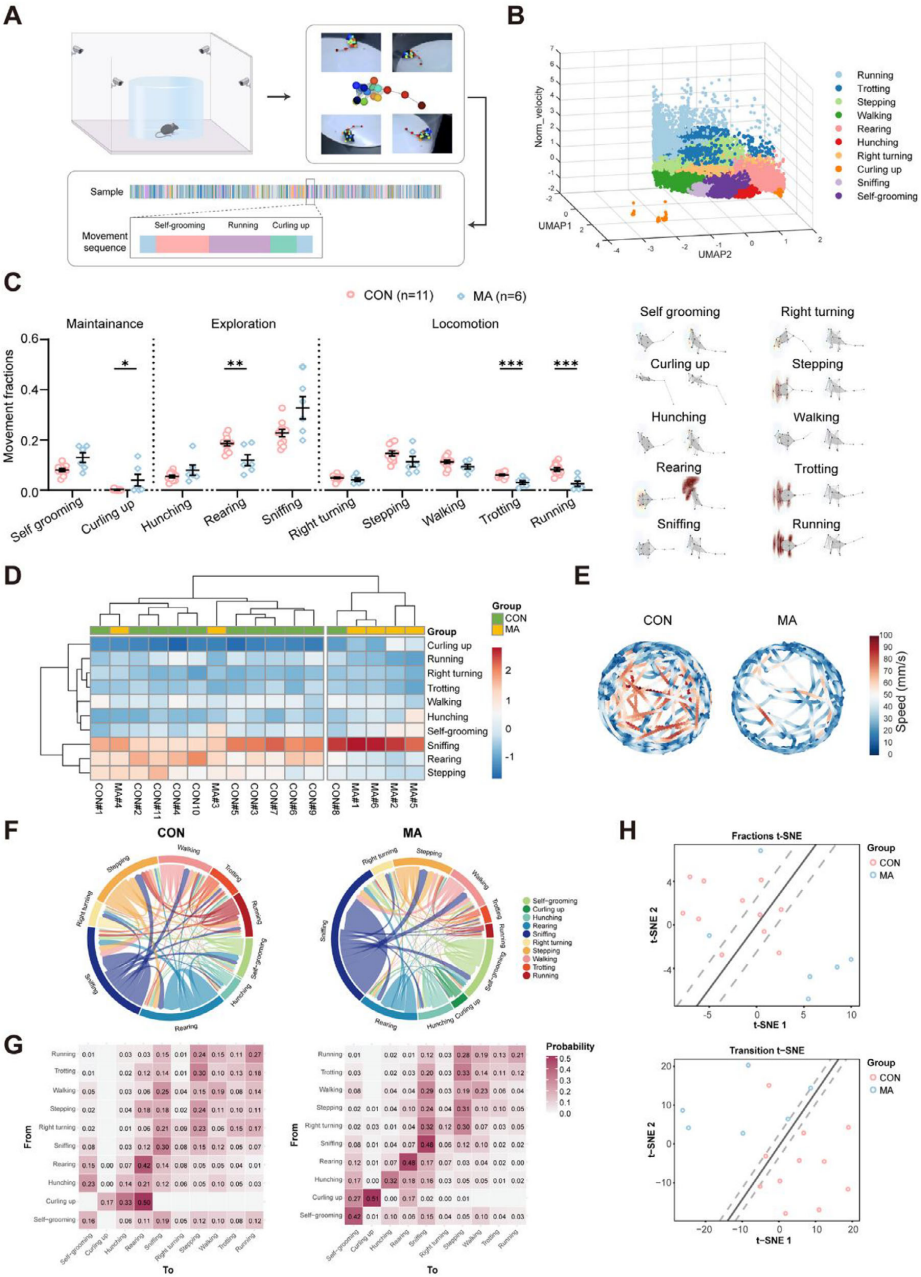
Differences in Spontaneous Behaviors between CON and MA Group Mice. A) The skeletal trajectories of mice moving freely in the open field for 10 min were captured and reconstructed by four cameras, and the actions were classified using an unsupervised algorithm. B) UMAP dimensionality reduction plot of action segments of all samples in three‐dimensional space. C) (Left) Results of differential analysis of ten types of actions in MA (n = 6) and CON (n = 11) group mice; (Right) Average skeletal heat map of ten types of actions. D) Hierarchical clustering heat map of action proportions (Clusters = 2). E) Plot of average velocity trajectories for each group. F) Action transition probabilities and G) chord diagrams. On the left is the CON group, and on the right is the MA group. In these diagrams, the arc length of each action type represents its average proportion within the group. The direction and size of the arrows between two action types indicate the direction and probability of action transitions. H) 2D t‐SNE dimensionality reduction results plots of action proportions (top) and action transition probabilities (bottom), along with the decision boundaries of the SVM classifier. ^*^P < 0.05, ^**^P < 0.01, ^***^P < 0.001, by Two‐tailed unpaired separate variance estimation t‐test, Two‐tailed unpaired t test and Mann‐Whitney U test. Data are presented as the mean ± SEM.

To systematically characterize potential alterations in spontaneous behaviors within the MA model, we used a parallel multi‐layered 3D motion capture framework that preserves hierarchical behavioral dynamics. Using this approach, we performed a quantitative analysis of the cross‐scale behavioral patterns in both the MA and CON groups using continuous multiview kinematic monitoring (**Figure** **3**A). We manually grouped these 40 behavioral patterns and obtained 10 main types of behaviors from 17 animals: running, trotting, stepping, walking, rearing, hunching, right turning, curling‐up, sniffing, and self‐grooming (Figure 3B, Figure S3A, Supporting Information). These behaviors are classified into three major categories according to their movement patterns: maintenance, exploration, and locomotion. In the maintenance category, the MA group exhibited more curling behavior than the CON group. Regarding exploration, the rearing of the MA group significantly decreased. The proportion of locomotor behaviors in the MA group showed a decline; specifically, trotting and running actions were significantly lower than those in the CON group. Overall, compared with the CON group, the mice in the MA group were less willing to explore the novel environment and often assumed a curled‐up defensive posture, which also reflected their anxious emotions (Figure 3C, Figure S3B–E, Supporting Information). The hierarchical clustering results of the action proportion showed that the behavioral patterns of the four samples in the MA group and one sample in the CON group were relatively consistent. Many sniffing actions formed one cluster. In the other cluster, there were generally fewer crouching behaviors and more sniffing, rearing, and stepping actions, and most of the samples belonged to the CON group (Figure 3D). The heatmap of the average speed trajectories for each group also showed that the movement speed and displacement distance of the MA group were lower than those of the CON group (Figure 3E, Figure S4A–C, Supporting Information). The chord diagram and heatmap indicated that in the CON group, there were relatively more self‐transitions in the rearing and sniffing actions. In addition, half of the curling‐up actions transitioned to rearing actions, suggesting a positive exploratory attitude in the CON group. In contrast, in the MA group, most actions were self‐transitions, including self‐grooming, curling‐up, hunching, rearing, and sniffing, implying a tendency for the MA group to maintain existing actions (Figure 3F‐G). To compare the behavioral patterns of the MA and CON groups, we used the t‐distributed stochastic neighbor embedding method to reduce the dimensionality of the samples using the action proportion and action transition probability data (Figure 3H, Figure S4D, Supporting Information). In the dimensionality reduction results of the action proportions, the linear classifier (support vector machine) could classify the CON and MA groups to a certain extent. Classification based on action transition probabilities was more effective, indicating significant differences in the behavioral patterns between the CON and MA groups, particularly in the tendencies of action transitions. Collectively, our multiscale behavioral analysis revealed that MA modeling reduced exploratory drive, increased defensive posturing, and disrupted behavioral sequencing patterns, demonstrating a fundamental reorganization of spontaneous behavioral hierarchies that parallels clinical anxiety‐related comorbidities in chronic pain conditions.

To explore whether the MA model differs from existing trigeminal or limb injury paradigms, we conducted a systematic comparison between MA‐induced pain and trigeminal neuralgia. Our results demonstrate that the MA model exhibits distinct phenotypic features that differentiate it from classical trigeminal neuropathic pain models, containing different pain localization, food consumption, and facial parameters. In detail, the pain localization in the trigeminal nerve injury model was widespread, while that in the MA model were more localized to the temporomandibular joint (Figure S5A‐C, Supporting Information). Besides, the MA mice showed more severe feeding suppression than that of trigeminal nerve injury mice, which may be attributed to the impairment of masticatory function caused by mandibular asymmetry (Figure S5D, Supporting Information). Regarding facial parameters, the ear position of MA mice was more forward, while the ear angle was more straight. The degree of face inclination in the MA mice was flatter (Figure S5E‐K, Supporting Information). These data highlight that the MA model shows TMD‐specific pathophysiology.

### 2.2 Whole‐Brain FOS Activation Screening in MA Model Mice

**Figure 4:**
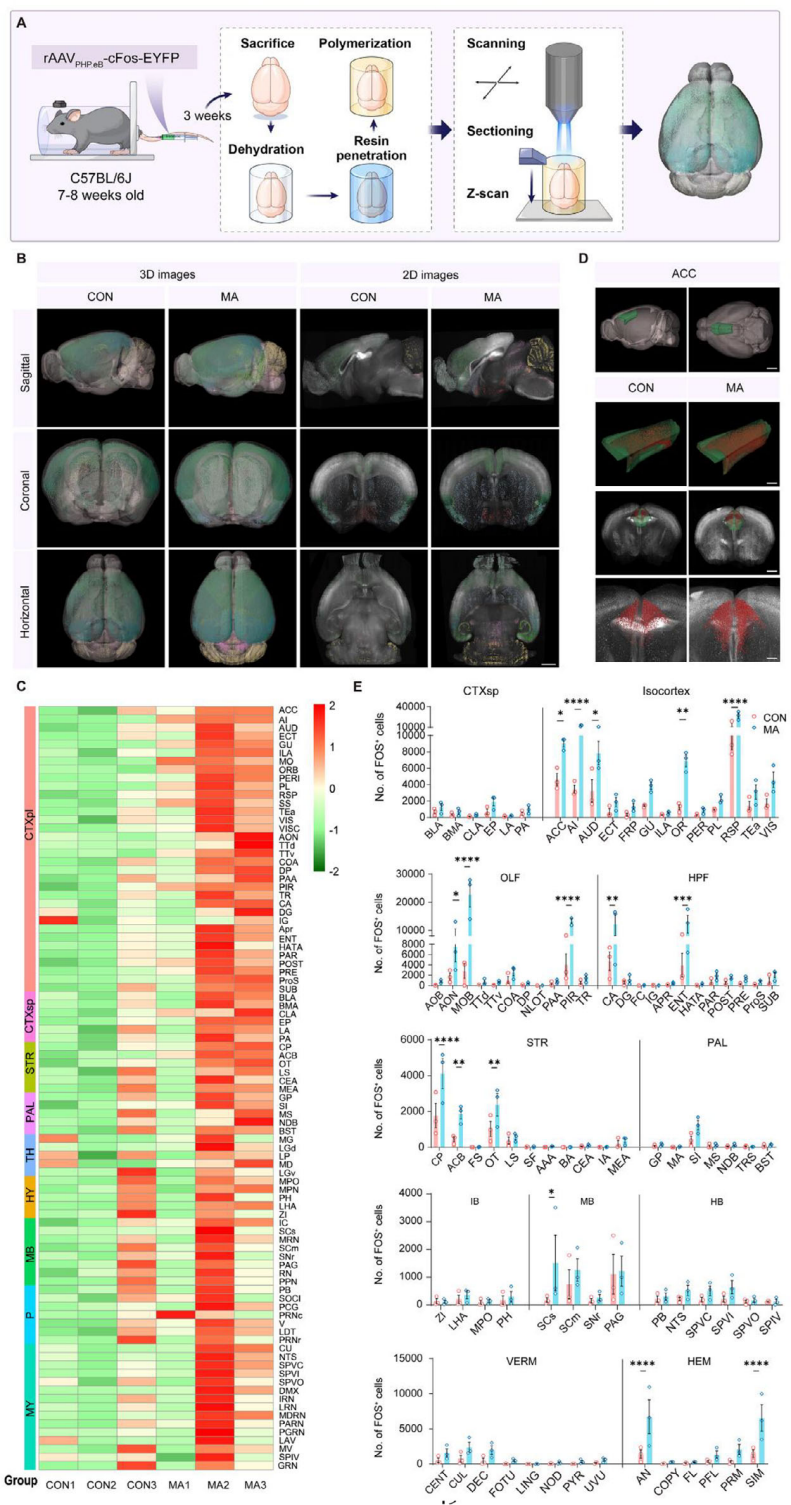
Brain‐wide mapping of FOS expression with fluorescence micro‐optical sectioning tomography in MA mouse model. A) Experimental flowchart of whole‐brain mapping of FOS expression using fluorescence micro‐optical sectioning tomography. B) Representative images of c‐Fos expression at 3D and 2D levels, including sagittal, coronal, and horizontal planes. Scale bars: 1000 µm. C) Heatmaps showing FOS expression in various brain regions (n = 3). D) Representative images of FOS expression in the ACC in different perspectives. Scale bars: 1000, 500, 1000, 500 µm (from top to bottom). E) Statistical analysis in c‐Fos expression between the CON and MA groups across distinct brain regions (n = 3). ^*^P < 0.05, ^**^P < 0.01, ^***^P < 0.001, ^****^P < 0.0001, by Kruskal‐Wallis H test, One‐way ANOVA, Welch ANOVA, *post hoc* Dunn multiple comparison, Tukey multiple comparison, and Dunnett T multiple comparison. Data are presented as the mean ± SEM.

Previous studies have reported the effects of chronic orofacial pain in multiple brain regions using neuroimaging techniques.^[^ ^24^, ^25^ ^]^ However, research on the effects of MA on the entire brain is still lacking. To map brain‐wide neuronal activation in MA mice, we systemically administered AAV_PHP.eB_‐cFos‐EYFP to label activity‐dependent neurons, followed by whole‐brain imaging using the fMOST. This approach enabled a quantitative analysis of cumulative neural activation patterns across the entire brain in the MA and CON groups (**Figure** **4**A–C). 3D reconstruction across the sagittal, coronal, and horizontal planes revealed significantly increased FOS‐EYFP^+^ neuron counts in multiple brain regions of MA mice compared with controls, including the ACC, demonstrating widespread neuronal activation in this chronic pain model (Figure 4D, Video S2, Supporting Information). In particular, the density of FOS‐EYFP^+^ neurons increased in the ACC, cortical plate, cortical subplate, striatum, pallidum, thalamus (TH), midbrain, and parietal lobe (Figure 4E). Among these significantly altered brain regions, the ACC demonstrated one of the most robust activation patterns, consistent with its established role in pain and anxiety‐related behaviors.^[^ ^26^, ^27^ ^]^ To systematically identify whether the ACC is a key brain region responsive to MA modeling, we analyzed the brain‐wide activation maps using linear support vector classification and calculated the corresponding activation patterns to discriminate between CON and MA groups in an unbiased manner.^[^ ^28^, ^29^ ^]^ The activation pattern heatmap, values of classifier weights, the feature covariance matrix, as well as the curve of cumulative activation contribution between the CON and MA mice indicated that the ACC is one of the predominant contributors in the discrimination between the CON and MA mice (Figure S6A‐D, Supporting Information). In addition to the ACC region, compared with the CON group, several other brain regions were significantly activated due to the establishment of the MA model, such as the somatosensory cortex (SS), anterior insula (AI), auditory cortex (AUD), orbitofrontal cortex (ORB), and retrosplenial cortex (RSP) (Figure S7A‐E, Supporting Information).

**Figure 5:**
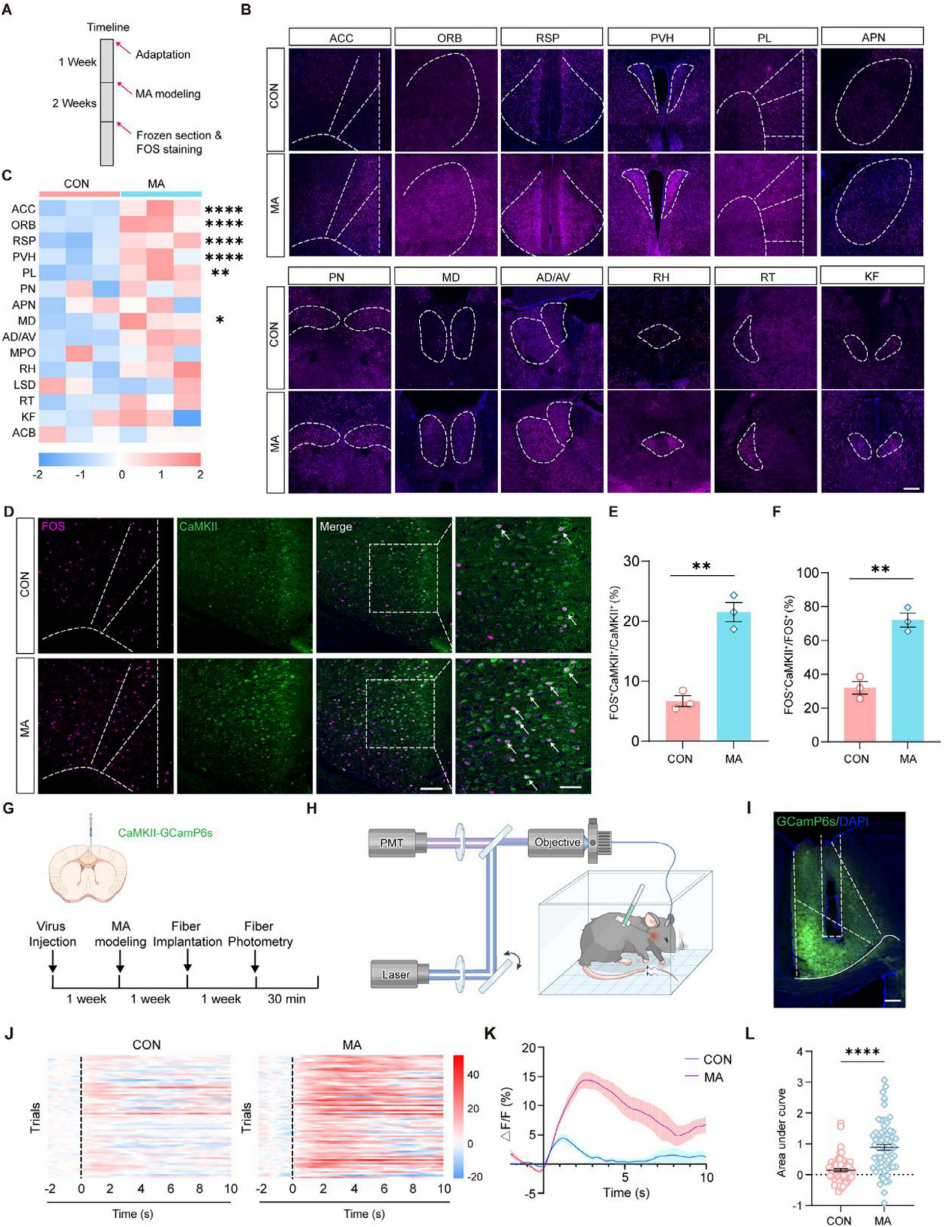
Whole‐brain FOS mapping and the role of CaMKII neurons in the ACC of MA model mice. A) Timeline of MA Modeling and FOS Staining. B) Representative images of FOS staining in various brain regions for CON and MA groups. Dashed lines outline regions of interest, highlighting differences in FOS expression. Scale bars: 200 µm. C) Heatmap illustrating FOS expression levels in different brain regions for CON and MA groups. Color‐coding represents expression intensity, with asterisks indicating statistical significance (n = 3). D) Immunofluorescence images showing co‐labeling staining of FOS (magenta) and CaMKII (green) in the brain. Merged images highlight co‐localization (arrows) in CON and MA groups, with dashed lines marking regions of interest. Scale bars: 100 and 500 µm (enlarged insets). E,F) Bar graphs quantifying the percentage of FOS‐positive cells co‐expressing CaMKII (E) and CaMKII‐positive cells co‐expressing FOS (F) in CON and MA groups denote significant differences (n = 3). G) The timeline of injecting the CaMKII‐GCaMP6s virus, establishing the MA model, implanting optical fibers, and conducting fiber photometry for real‐time monitoring of neural activity. H) Diagram of the fiber photometry system, featuring components such as laser, PMT (photomultiplier tube), and objective, for detecting neural activity in freely‐moving mice. I) Image showing GCaMP6s (green) expression in the brain, counterstained with DAPI (blue), highlighting the area for in vivo imaging analysis. Scale bars: 200 µm. J) Heatmaps of neural activity traces (ΔF/F) over time for CON and MA groups, with each row representing an individual trial. Color scale indicates the magnitude of activity change. K) Graphs showing the average neural activity profiles over time for CON and MA groups, highlighting distinct activity patterns. L) Bar graph comparing the area under the curve of neural activity between CON and MA groups (n = 66 (CON), n = 72 (MA)). ^*^P < 0.05, ^**^P < 0.01, ^****^P < 0.0001, Two‐way ANOVA, two‐tailed unpaired t test, *post hoc* Šídák multiple comparison. Data are presented as the mean ± SEM.

To verify the fMOST results, we used immunofluorescence staining to map the expression pattern of FOS throughout the brain in the MA model (**Figure** **5**A). Consistent with the fMOST results, compared with the CON group, the MA group showed more abundant FOS expression in many brain regions, including the ACC, ORB, RSP, paraventricular hypothalamus (PVH), prelimbic cortex (PL), and mediodorsal thalamus (MD) (Figure 5B,C). Focusing on the ACC as a representative region showing robust activation changes, we performed a cell type‐specific analysis and observed significantly enhanced co‐localization of FOS with CaMKII^+^ neurons in MA mice compared with controls. This preferential activation of CaMKII‐expressing projection neurons suggests their potential role in mediating MA‐related circuitry (Figure 5D–F). To further investigate the functional changes in CaMKII^+^ neurons in the ACC within the MA group in vivo, following the rAAV (Recombinant adeno‐associated virus)‐mediated expression of GCaMP6s in ACC CaMKII^+^ neurons, we acquired calcium signals during graded von Frey filament stimulation using fiber photometry (Figure 5G–I). We found that under the same level of nociceptive stimulation, the ACC CaMKII^+^ neurons in MA mice exhibited significantly amplified calcium responses compared with controls (Figure 5J–L). These findings suggest that the MA model induces widespread brain activation, with a particularly robust and selective engagement of ACC CaMKII^+^ neurons, which show both elevated baseline activity and amplified evoked responses.

### 2.3 Integrated Multi‐Omics Analysis Reveals Altered Gene Expression, Cell–Cell Interactions, and Signaling Pathway Dynamics in the Anterior Cingulate Cortex of MA Model Mice

**Figure 6:**
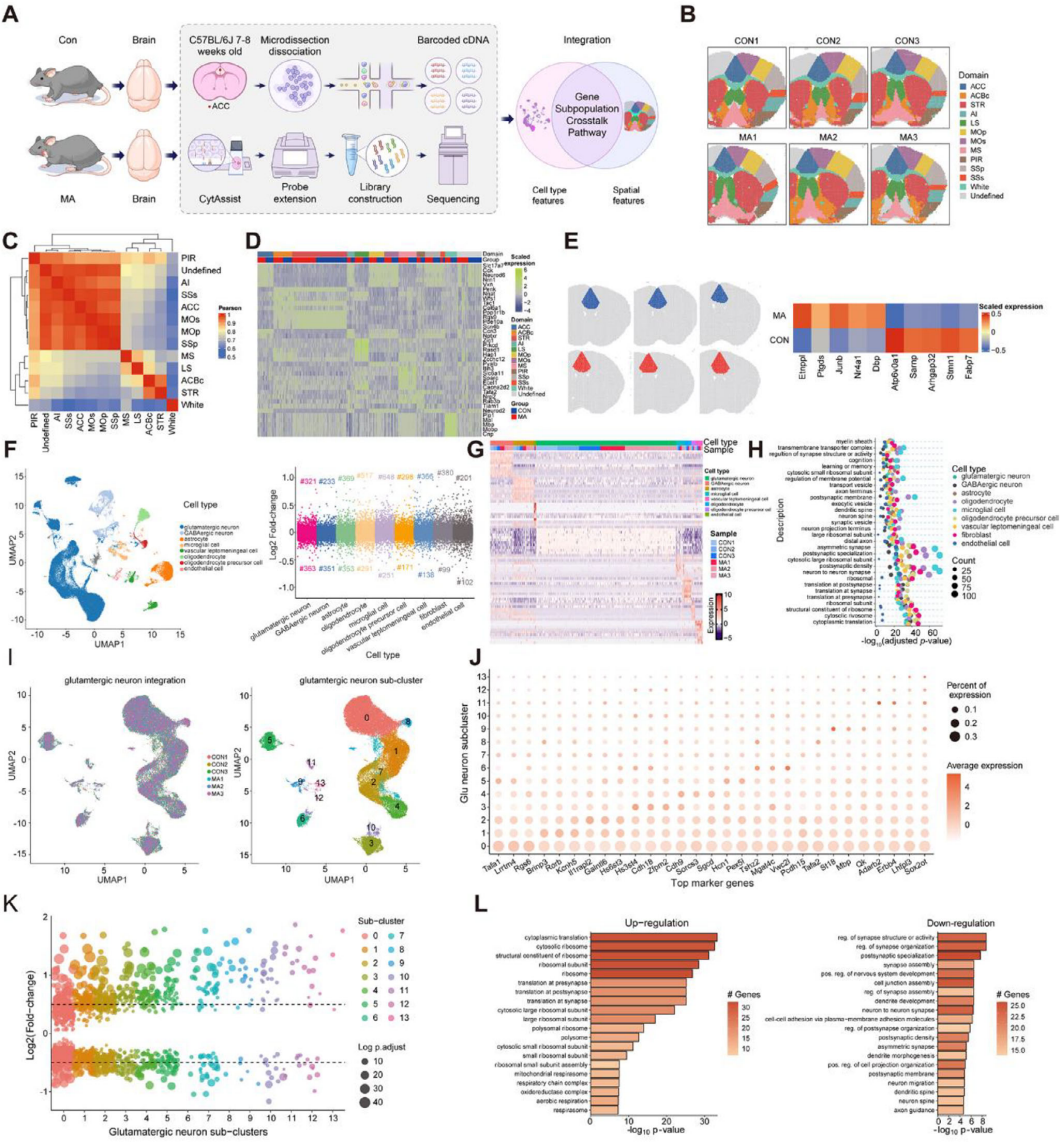
Combined analysis of spatial transcriptomics and single‐cell sequencing of the ACC in mice of the CON and MA groups. A) Schematic diagram of the combined analysis of single‐cell and spatial transcriptomics (spatial‐omics). B) Anatomical overview of the spatial point clusters in the brain slices of the CON and MA groups (n = 3). C) Heatmap showing the similarity of gene expression among spatial clusters. D) Heatmap showing the genes enriched in each spatial cluster. E) Spatial transcriptomics reveals the top 5 upregulated and downregulated genes in the ACC brain region compared with the CON group. F) T‐SNE (t‐distributed Stochastic Neighbor Embedding) plot showing the cell types identified in the single‐cell results of the CON and MA groups and the number of differentially expressed genes (upregulated versus downregulated) for each cell type (n = 3). G) The TOP 10 genes most enriched in each cell population identified by single‐nucleus RNA sequencing (snRNA‐seq). H) Pathway enrichment analysis of the genes in each cell population identified by snRNA‐seq. I) t‐SNE plot of each cluster of excitatory neurons. J) The top three genes enriched in each subgroup (cluster). K) The number of differentially expressed genes in each subgroup. L) Diagram of the enriched pathways of excitatory neurons identified by single‐cell sequencing.

To obtain a more comprehensive gene expression atlas depicting the spatial distribution and cellular specificity of gene expression in the ACC of mice in the CON and MA groups, we performed a combined analysis of spatial transcriptomics (ST) and single‐cell sequencing data (**Figure** **6**A). Using the Seurat R package, we identified 12 major ST spot clusters in the spatial transcriptomic data (Figure 6B). Each spot represented a sampling point with fixed coordinates in the tissue section. These 12 clusters exhibited regional specificity and corresponded to different neuroanatomical regions of the brain tissue. The heatmap showed a higher degree of similarity and closer distribution among the ACC, secondary motor cortex, and primary motor cortex (Figure 6C). We presented the top enriched genes in these clusters using a heatmap and compared them with the remaining clusters (Figure 6D). Using the Loupe local discriminant algorithm, we found that compared with the CON group, the expression levels of Etnppl, Ptdgs, Junb, Nr4a1, and Dbp were upregulated in the ACC of the MA group. However, the expressions of Atp6v0a1, Sarnp, Arhgap32, Stmn1, and Fabp7 were downregulated in the ACC of the MA group (Figure 6E). We sequenced and analyzed 33,201 and 39,120 cell nuclei from the ACC of mice in the CON and MA groups, respectively. Using previously reported marker genes, we annotated nine major cell cluster subtypes: glutamatergic neurons, GABAergic inhibitory neurons, astrocytes, oligodendrocytes, microglial cells, oligodendrocyte precursor cells, leptomeningeal cells, fibroblasts, and endothelial cells (Figure 6F, Figure S8C, Supporting Information). We verified the accuracy of cluster differentiation using differentially expressed genes (Figure S8D,E, Supporting Information). Among these nine cell cluster subtypes, the number of upregulated and downregulated genes varied across the different clusters. Next, we highlighted the top five enriched genes in each cluster using a heatmap and compared them with those in the remaining clusters (Figure 6G‐H). These genes may serve as markers of different cell types. We further divided the glutamatergic neurons into 14 cell subpopulations (Figure 6I). The specific expression of these markers in their respective neuronal types, identified through single‐nucleus RNA sequencing (snRNA‐seq) data, indicated the accuracy and high quality of our dataset. Subsequently, we presented the top three enriched genes in each glutamatergic cell subpopulation using a bubble plot and compared them with those in the remaining clusters (Figure 6J). We found that the upregulation and downregulation patterns of genes varied among the different subpopulations of glutamatergic neurons (Figure 6K). Moreover, pathway enrichment analysis of glutamatergic neurons revealed that the expression of key pathways related to ribosomes and protein synthesis was upregulated, mainly including cytoplasmic translation, cytosolic ribosomes, and structural constituents of the ribosomes. However, pathways related to synaptic function, such as synaptic structure or activity, synaptic organization, and postsynaptic specialization, were significantly downregulated (Figure 6L). To investigate the spatial distribution characteristics of cell subsets, based on our snRNA‐seq and ST data, the CellTrek analysis method was applied to directly map single cells back to their spatial coordinates in brain sections (Figure S8A,B, Supporting Information). Compared with the CON group, the expression distribution patterns of glutamatergic neurons, GABAergic neurons, and oligodendrocyte precursor cells were different in the MA group. Finally, we explored the expression enrichment and spatial distribution of the marker genes of glutamatergic neurons belonging to 14 subpopulations (Figure S9A,B, Supporting Information).

**Figure 7:**
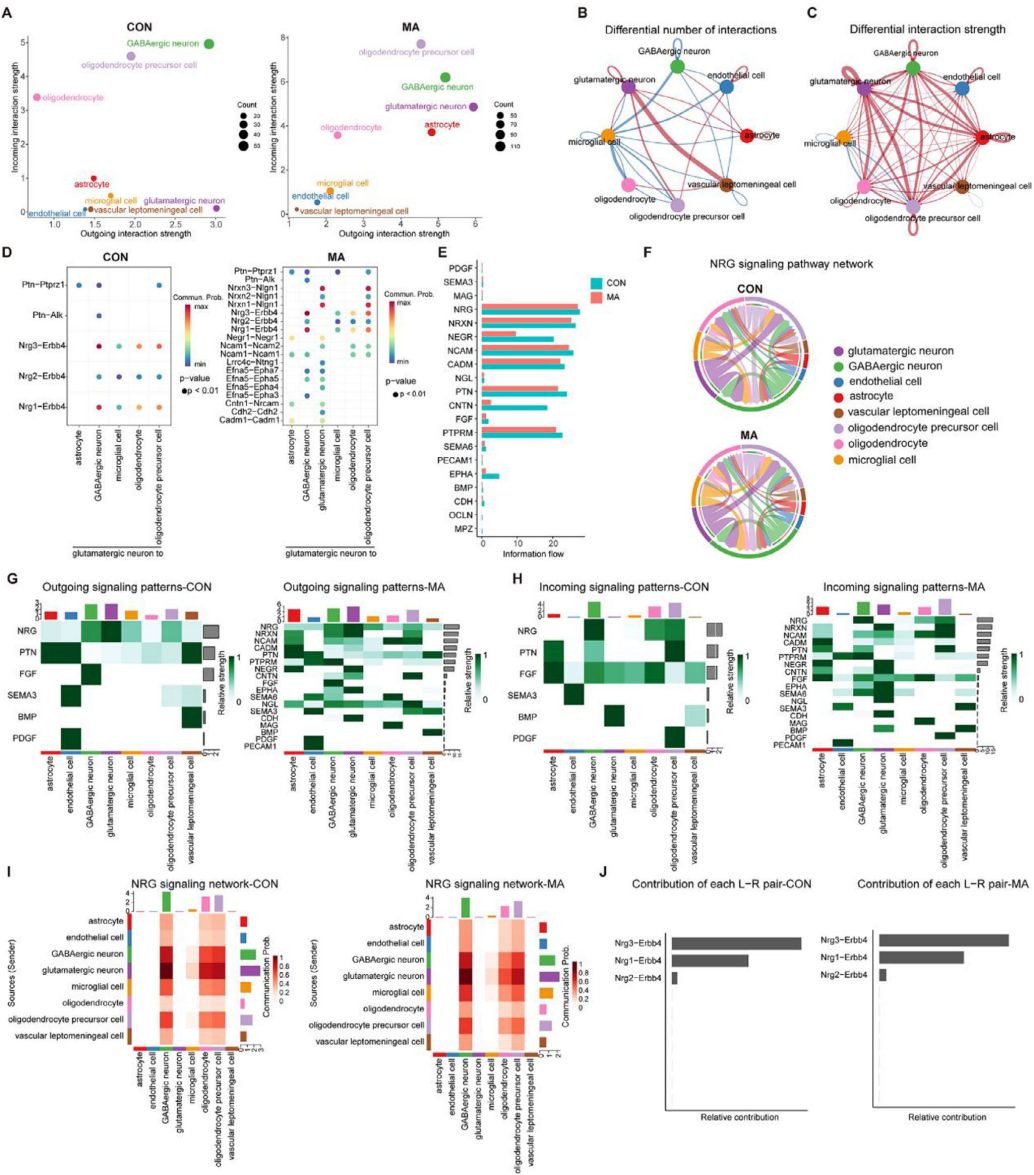
Differences in cell interactions and cell signaling pathways between the CON and MA groups. A) The intensity of afferent and efferent signals of cells in each cluster from single‐cell sequencing of both the CON and the MA groups. B) Analysis of the number of differentially expressed genes related to cell interaction. C) Analysis of the differential intensity of cell interaction. D) The expression differences of ligand‐receptor pairs derived from glutamatergic neurons between cells of the CON and MA groups. E) Changes in signaling pathways (Information flow). F) The NRG signaling pathway network between cells of the CON and MA groups (mainly consisting of glutamatergic neurons). G‐H) Schematic diagram of the outgoing and incoming signal patterns between various cells in the CON and MA groups. I) Heatmap of the NRG signals between cells of the CON and MA groups. J) Diagram of the contribution of ligand‐receptor pairs in the NRG pathway between cells of the CON and MA groups.

In single‐cell sequencing of the CON and MA groups, the intensities of incoming and outgoing signals varied among different cell clusters (such as glutamatergic neurons, GABAergic neurons, and oligodendrocytes). Moreover, both the incoming and outgoing signal intensities of the glutamatergic neurons in the MA group were higher than those in the CON group (**Figure** **7**A). To comprehensively explore the interaction landscape among various cell clusters in the CON and MA groups, we performed a communication analysis using the CellChat tool based on single‐cell sequencing data of ACC neurons and spatial transcriptomic data (Figure 7B). The results showed that, compared with the CON group, the cell interactions between glutamatergic neurons and vascular leptomeningeal cells caused by differential gene expression were significantly decreased in the MA group (Figure 7B, Figure S10A, Supporting Information). However, compared with the CON group, the cell interactions between glutamatergic neurons and astrocytes in the MA group increased, and the interactions among glutamatergic neurons themselves also significantly increased (Figure 7C, Figure S10B, Supporting Information). The heatmap and histogram present results consistent with those described above (Figure S10C,D, Supporting Information). We used the ST dataset to determine the spatial coordinates of the cell–cell interactions. By integrating the snRNA‐seq data, we identified the specific cells involved in these interactions. Using stLearn, we calculated the spatial co‐expression of ligand‐receptor (L‐R) genes within and between ST spots in the ACC and successfully identified several valid L‐R pairs. Compared with the MA group, glutamatergic neurons in the CON group showed a more enriched expression of L‐R pairs with other clusters. Among these, Nrxn3‐Nlgn1 (between glutamatergic neurons) were highly expressed, which are involved in the regulation of synaptic stability and plasticity (Figure 7D).^[^ ^30^ ^]^ Moreover, compared with the CON group, the expression of L‐R pairs between GABAergic neurons and other clusters in the MA group was also more abundant (Figure S10E, Supporting Information). The L‐R pairs between astrocytes and the cells of the remaining clusters also underwent changes (Figure S10F, Supporting Information). We constructed signaling pathway networks for NRG (Figure 7E,F), PTN, and SEMA3 (Figure S10G, Supporting Information). The heatmap demonstrated that, when compared with the CON group, the input and output signal patterns in the MA group were significantly more abundant. Additionally, it was observed that the NRG signaling pathway was highly enriched in both the input and output signals of glutamatergic neurons in the CON group. However, in the output signal patterns of the MA group, there was a remarkable reduction, which suggests that the establishment of the MA model has led to alterations in synaptic regulatory functions and synaptic plasticity (Figure 7G,H). Moreover, we compared the differences in the enrichment levels of the NRG (Figure 7I), PTN (Figure S10H, Supporting Information), and SEMA3 (Figure S10K, Supporting Information) signaling pathways among various clusters of neurons between the CON and MA groups using a heatmap. Furthermore, we compared the differences in the contributions of the L‐R pairs related to the NRG (Figure 7J), PTN (Figure S10I,J, Supporting Information), and SEMA3 (Figure S10L,M, Supporting Information) to the signaling pathways between the CON and MA groups.

### 2.4 Structural and Functional Plasticity of ACC Excitatory Synapses in the MA Model

**Figure 8:**
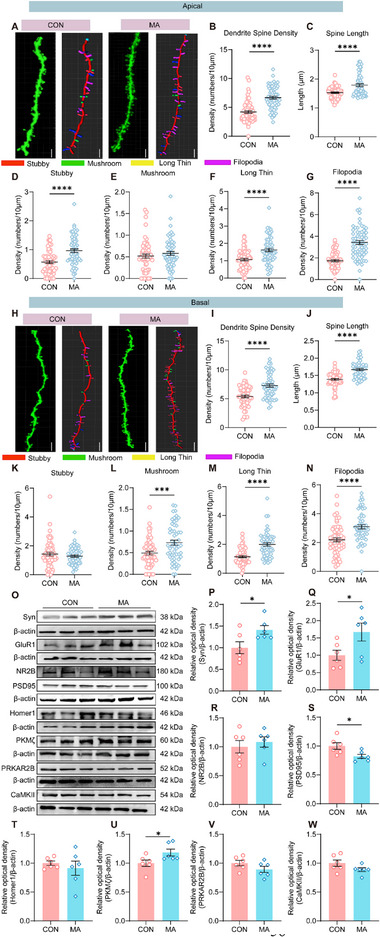
Structural and functional plasticity of ACC excitatory synapses in the MA model. A) Representative confocal images showing different types of dendritic spines (stubby in red, mushroom in green, long thin in yellow, filopodia in magenta) in CON and MA groups. Scale bars: 5 µm. B) Quantification of dendrite spine density, revealing a significant increase in the MA (n = 60) group compared to CON (n = 62). C) Spine length measurement, with MA treatment leading to a notable elongation (n = 56 (MA), n = 60 (CON)). D‐G) Density analysis of specific spine subtypes (stubby, mushroom, long thin, filopodia), all showing marked elevation in the MA group (n = 55 (CON), n = 61 (MA)). H) Representative confocal images of dendritic spines in CON and MA groups, labeled by spine type. I) Dendrite spine density is significantly higher in the MA group (n = 59 (CON), n = 54 (MA)). Scale bars: 5 µm. J) Spine length is increased following MA treatment (n =59 (CON), n = 54 (MA)). K‐N) Density of individual spine subtypes (stubby, mushroom, long thin, filopodia) is augmented in the MA‐treated samples (n = 54–59). ^***^P < 0.001, ^****^P < 0.0001, by Two‐tailed unpaired t test. O) Representative western blotting bands for synaptic proteins including Syn, GluR1, NR2B, PSD95, Homer1, PKMζ, PRKAR2B, CaMKII, and β‐actin in the CON and MA groups. P‐W) Quantitative analyses of the relative optical densities of these proteins normalized to β‐actin. Compared with the CON group, the MA group shows significantly increased expressions of Syn, GluR1, and PKMζ, along with a marked decrease in PSD95 expression (n = 6). ^*^P < 0.05, ^***^P < 0.001, ^****^P < 0.0001, by Two‐tailed unpaired t test, Welch‘s t test, Mann‐Whitney U test. Data are presented as the mean ± SEM.

We further determined whether MA altered the synaptic structure and function of CaMKII^+^ neurons in the ACC. First, we detected morphological changes in the apical and basal dendrites of CaMKII^+^ neurons in the ACC of mice in the MA and CON groups by retro‐orbital injection of the AAV_PhP.eB_‐hSyn‐EGFP‐P2A‐EGFPf‐WPRE‐HGHpA, which sparsely labeled neurons with enhanced green fluorescent protein (EGFP) (**Figure** **8**A). In the apical dendrites, compared with the CON group, the ACC pyramidal neurons in the layer II/III of ACC exhibited an increase in the density and length of spines in the MA group, along with a higher abundance of stubby‐shaped spines, long thin‐shaped spines, and filopodia (Figure 8B–G). In the basal dendrites, the density and length of dendritic spines increased, and there was an increased number of mushrooms, long thin‐shaped spines, and filopodia (Figure 8H–N). Then, we measured the molecular basis of synaptic changes via western blotting. Compared with the CON group, the expressions of Syn (synaptophysin), GluR1 (Glutamate Ionotropic Receptor AMPA Type Subunit 1), and PKMζ (protein kinase M zeta) in mice of the MA group were significantly increased, while the expression of PSD95 (postsynaptic density protein 95) was significantly decreased. There were no significant changes in the expression of NR2B (glutamate ionotropic receptor NMDA type subunit 2B), Homer1, PRKAR2B (protein kinase cAMP‐dependent type II regulatory subunit beta) and CaMKII (Figure 8O‐W). To explore whether these plastic changes in the ACC are driven by pain, we administered gabapentin (i.p. injection, 100 mg/kg for 7 consecutive days) as an analgesic treatment to MA mice. The results showed that the anxiety‐like behaviors and hyperalgesia in the MA+saline mice (Figure S11A‐F, Supporting Information), as well as the FOS activation of ACC neurons (Figure S11G,H), Supporting Information were rescued after gabapentin injection. Western blotting analyses showed that the expression levels of Syn and GluR1 were significantly decreased compared with MA+saline group mice (Figure S11I‐N, Supporting Information). The above results indicate that the alterations in behavior and ACC plasticity in MA model mice are dependent on chronic pain.

**Figure 9:**
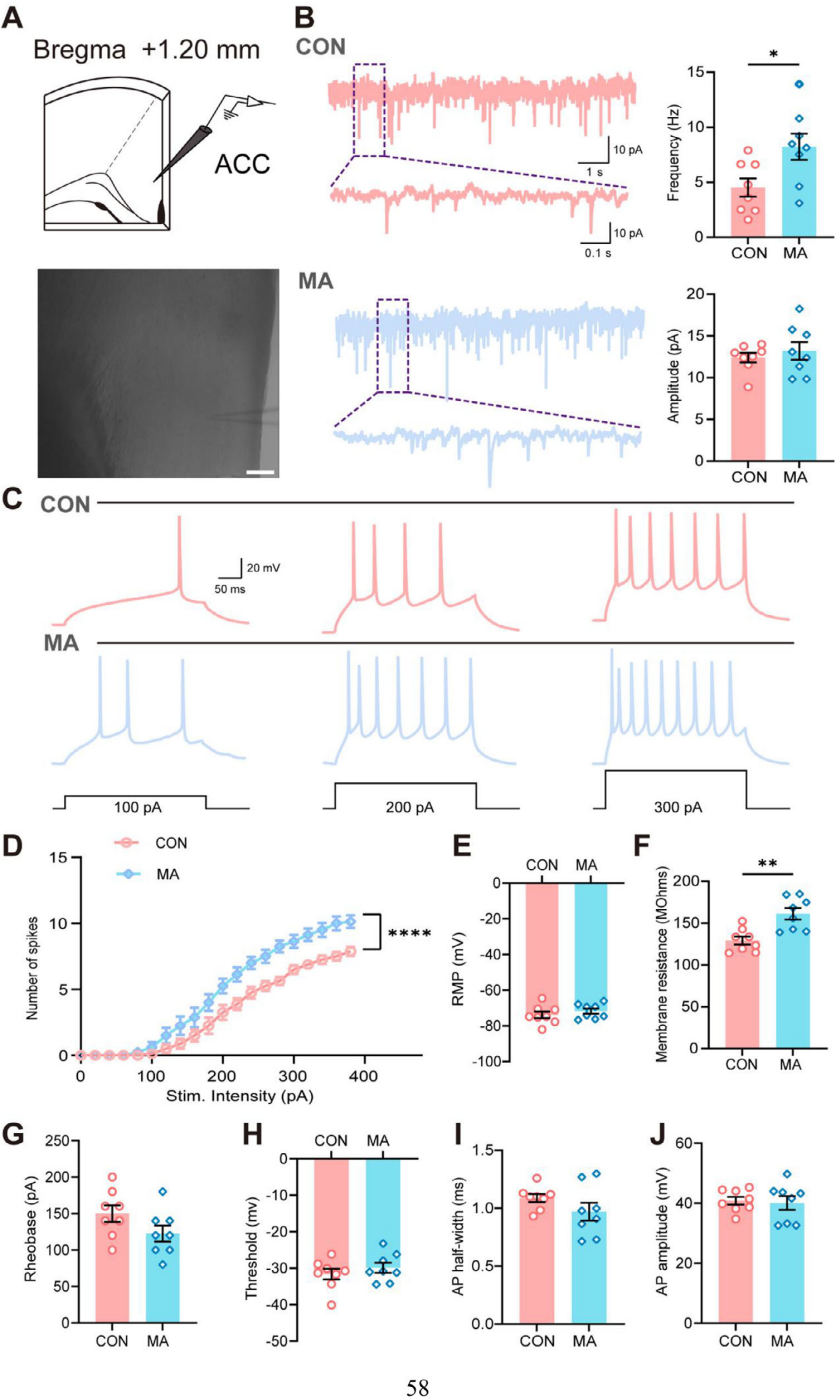
Effects of MA modeling on the excitatory synaptic transmission function of ACC neurons. A) Schematic illustration of the recording site in the ACC at Bregma +1.20 mm and a representative photomicrograph of the recording electrode placement. Scale bars: 100 µm. B) Representative traces of miniature excitatory postsynaptic currents (mEPSCs) in CON and MA groups, with quantification of mEPSC frequency (n = 8). C) Representative traces of action potential (AP) firing in response to different current injections (100, 200, 300 pA) in CON and MA neurons. D) A scatter line graph shows the changing trends of the number of neuronal action potential firings in the CON and MA groups under different stimulus intensities (n = 8). E–J) Bar charts display the comparison of various electrophysiological parameters between the CON group and the MA group. These parameters include resting membrane potential (RMP, E), membrane resistance (F), rheobase (G), action potential (AP) threshold (H), action potential half‐width (I), and action potential amplitude (J) (n = 8). ^*^P < 0.05, ^**^P < 0.01, ^****^P < 0.0001, Two‐tailed unpaired t test, Friedman M test. Data are presented as the mean ± SEM.

To examine the effect of functional changes on excitatory synaptic transmission following MA modeling, we performed whole‐cell patch‐clamp recordings of layer II/III neurons in the ACC (**Figure** **9**A). We found that, compared with the CON group mice, the frequency of α‐amino‐3‐hydroxy‐5‐methylisoxazole‐4‐propionic acid receptor (AMPAR)‐mediated spontaneous excitatory postsynaptic currents (sEPSCs) in the MA group was significantly higher, whereas there was no difference in the peak amplitude (Figure 9B). We also tested the neuronal excitability and membrane properties of the pyramidal neurons. We found that, with the same current injections, the number of action potentials in the MA group was significantly higher than that in the CON group (Figure 9C,D). Moreover, the membrane resistance of the MA group was significantly higher than that of the CON group (Figure 9E–J). These results indicate that MA model mice exhibit enhanced synaptic plasticity and hyperactivity in the ACC. Given the established role of the BLA‐ACC circuit in the comorbidity of chronic pain and anxiety,^[^ ^31^ ^]^ to verify whether the BLA is activated in MA model mice, we performed FOS staining and counting in the BLA. The results showed that compared with the CON group, the BLA in the MA group was significantly activated (Figure S12A,B, Supporting Information). Subsequently, we conducted optogenetics combined with electrophysiological recordings on the BLA‐ACC circuit. We first injected the optogenetic virus rAAV‐hSyn‐hChR2(H134R)‐EYFP into the BLA for 3 weeks, then we delivered 470 nm light stimulation to the ACC and performed recordings (Figure S12C, Supporting Information). The results demonstrated that the oEPSC (optically evoked excitatory postsynaptic current) in MA mice was significantly increased (Figure S12D,E, Supporting Information), while the paired‐pulse ratio was significantly decreased (Figure S12F,G, Supporting Information). This indicates that the probability of presynaptic neurotransmitter release in neurons of the BLA‐ACC circuit is enhanced in MA mice, accompanied by presynaptic overactivation of the BLA‐ACC pathway and consequent imbalance in excitatory transmission.

### 2.5 Inhibition of ACC CaMKII^+^ Neurons Alleviate Pain and Anxiety Comorbidities in the MA Model

**Figure 10:**
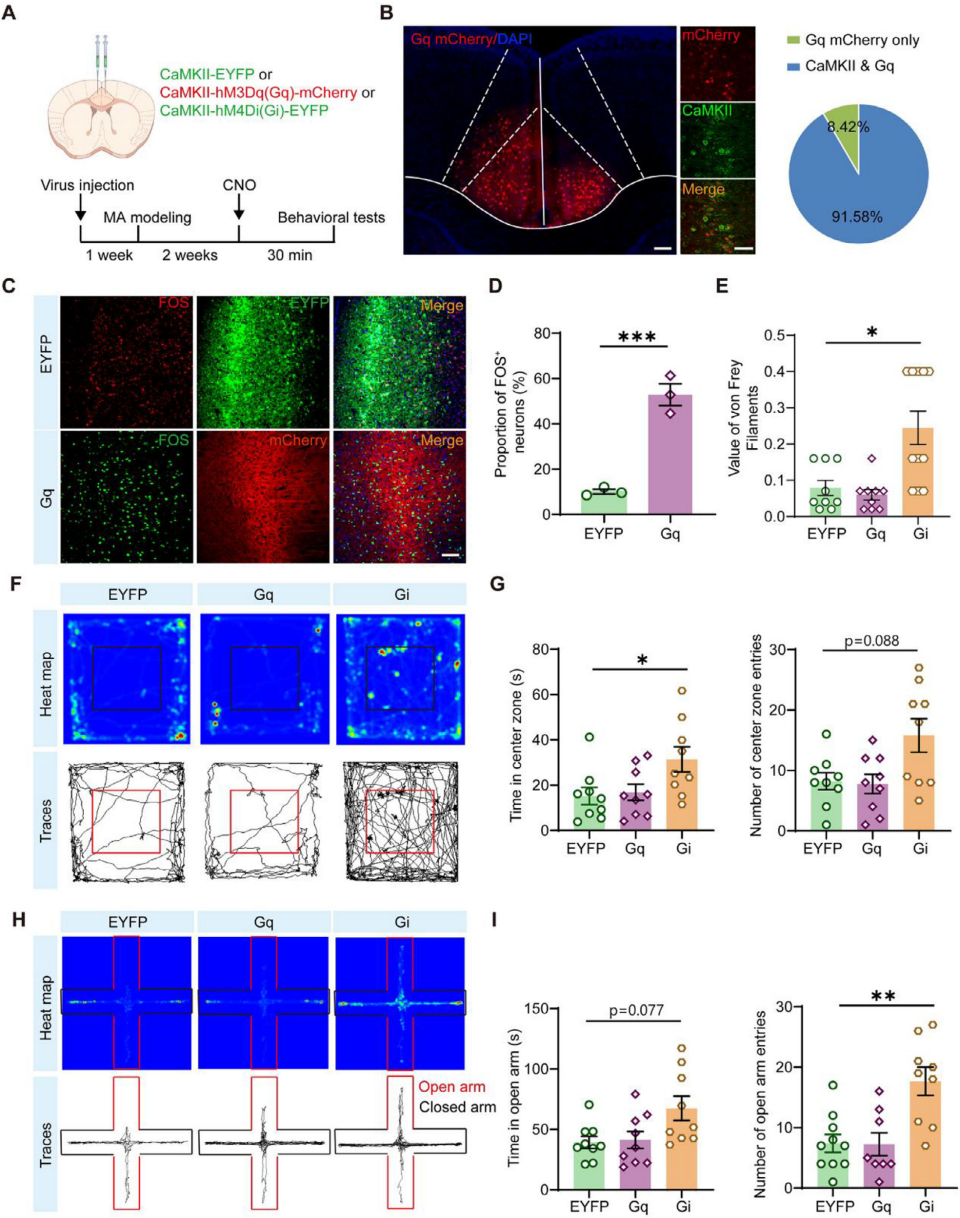
Chemogenetic regulation of CaMKII neurons in the ACC modulates hyperalgesia and anxiety‐like behaviors in male MA model mice. A) Schematic illustration of the experimental procedure, including virus injection into the brain, modeling of MA, treatment with CNO (clozapine‐N‐oxide), and subsequent behavioral tests. B) Representative images of immunofluorescence staining showing co‐localization of Gq (labeled with mCherry) and CaMKII in neurons, with DAPI counter‐staining for nuclei. Pie chart shows the proportion of cells expressing Gq mCherry only (8.42%) and those co‐expressing CaMKII and Gq (91.58%). Scale bars: 200 and 500 µm (enlarged insets). C) Immunofluorescence images of FOS expression (red) in neurons expressing EYFP or Gq (labeled with mCherry), with merged images demonstrating the co‐localization. Scale bars: 100 µm. D) Bar graph showing the proportion of FOS‐positive neurons, with a significant difference between EYFP and Gq groups (n = 3). E) Results of von Frey filaments test, indicating a significant difference in the value among EYFP, Gq, and Gi groups (n = 8–10). F) Heat maps and traces of animal movement in an arena, showing the distribution of movement for EYFP, Gq, and Gi groups. G) Bar graphs presenting the time spent in the center zone and the number of center zone entries, with significant differences between groups (n = 9). H) Representative heatmaps and trajectory plots of the elevated plus‐maze experiment in EYFP, Gq, and Gi groups. I) Bar graphs showing the time spent in open arms and the number of open arm entries, with significant differences among EYFP, Gq, and Gi groups (n = 9). ^*^P < 0.05, ^**^P < 0.01, ^***^P < 0.001, by Two‐tailed unpaired t test, One‐way ANOVA, Welch ANOVA, Kruskal‐Wallis H test, *post hoc* Tukey multiple comparison, Dunnett T multiple comparison. Data are presented as the mean ± SEM.

To evaluate the role of the ACC CaMKII^+^ neurons in the MA group, we injected rAAV‐CaMKII‐hM3Dq (Gq)‐mCherry‐WPRE‐hGHpA and rAAV‐CaMKII‐hM4Di (Gi)‐EGFP‐WPRE‐hGHpA into the ACC (**Figure** **10**A). Three weeks later, we activated and inhibited CaMKII^+^ neurons in the ACC following clozapine‐N‐oxide (CNO) administration. In addition, we verified the expression pattern of the chemogenic virus in CaMKII^+^ neurons using immunofluorescence staining (Figure 10B). Notably, rAAV‐CaMKII‐hM3Dq (Gq)‐mCherry‐infected neurons showed significantly higher co‐localization with FOS^+^ neurons than rAAV‐CaMKII‐EYFP, confirming its efficacy in targeting activated neuronal populations (Figure 10C,D).

Subsequently, the von Frey test was used to assess mechanical allodynia in the mice. We found that the pain threshold in the Gi group was significantly higher than that in the EYFP and Gq groups (Figure 10E). In the open field test, Gi mice spent more time in the center zone, whereas in the elevated plus‐maze, Gi mice showed higher entry counts (Figure 10F–I). Moreover, in the open field test, the total distance traveled by mice in the Gi group and the percentage of movement in the central area increased (Figure S13A, Supporting Information). In the elevated plus‐maze test, there was no significant difference in the total distance traveled by mice in the Gi group, whereas the percentage of movement in the open arms increased (Figure S13B, Supporting Information). Remarkably, chemogenetic inhibition of ACC CaMKII^+^ neurons in the ACC of female MA model mice also produced identical antinociception as well as anxiolysis effects (Figure S14A‐G, Supporting Information). Collectively, these findings indicate that suppressing ACC CaMKII^+^ neuronal activity effectively attenuates both mechanical allodynia and comorbid anxiety in the MA model. However, the role of the ACC and its downstream targets in MA remains to be explored in future studies.

## 3 Discussion

**Figure 11:**
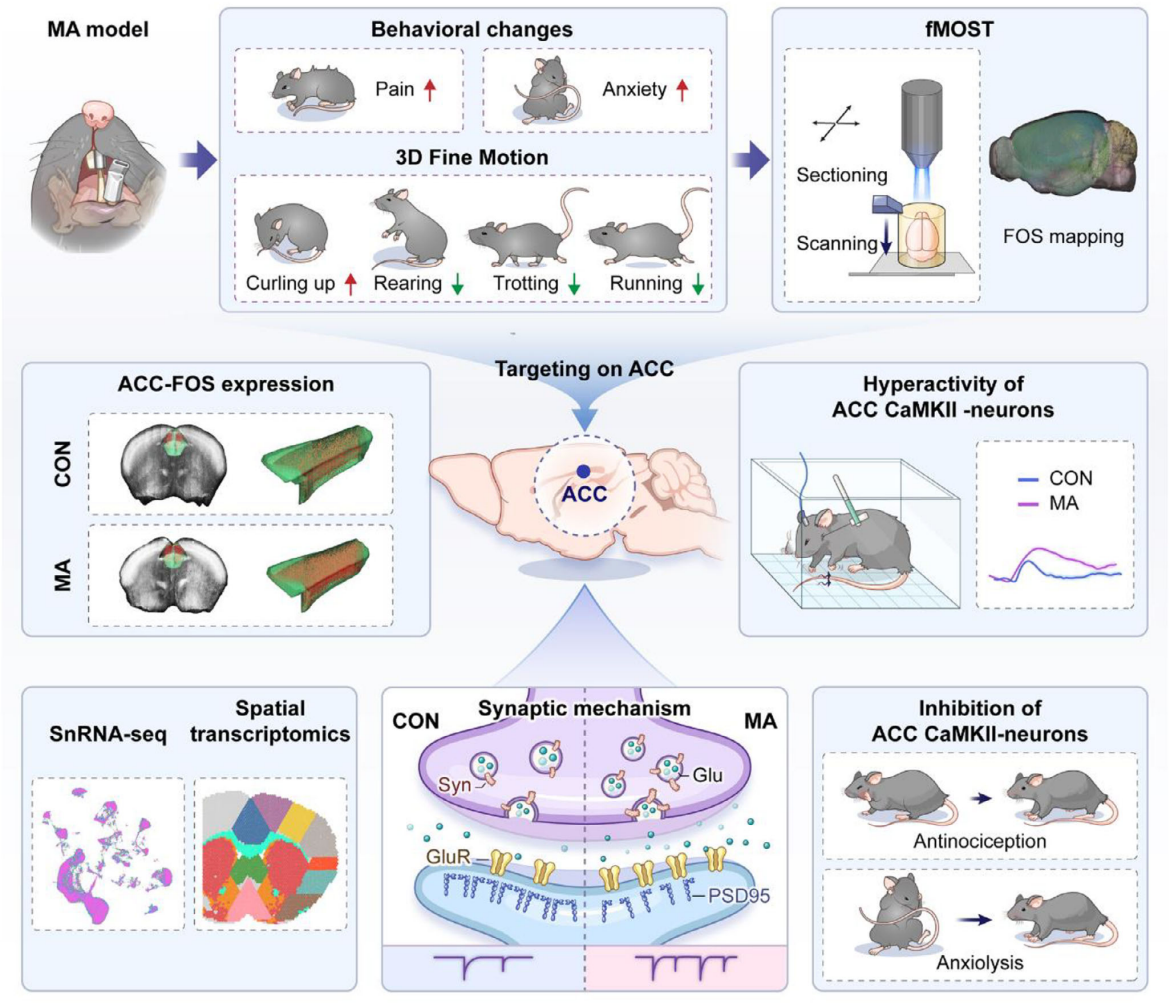
Schematic diagram of the underlying mechanism by which synaptic dysfunction in the anterior cingulate cortex underlies pain and anxiety in mandibular asymmetry model.

Emotional disorders accompanied by chronic orofacial pain severely affect physical and psychological health.^[^ ^32^, ^33^ ^]^ However, the underlying mechanism remains unclear. The present study aimed to clarify the role of the ACC neurons in orofacial pain in MA model mice. Using behavioral assays, we observed alterations in pain perception, anxiety‐like behaviors, and spontaneous behaviors in MA model mice. Through fMOST and FOS mapping, we identified hyperactivation of the ACC neurons. Subsequently, we employed a series of functional research techniques, including fiber photometry recording, multi‐omics analysis, western blotting, and electrophysiological recordings, to verify changes in the cell types and synaptic functions of the ACC neurons. Finally, using chemogenetics with the ACC as the target, we regulated the behavioral changes in the MA mouse model (Figure 11). Previous investigations have used a unilateral anterior crossbite (UAC) model to induce TMJ osteoarthritis.^[^ ^34^, ^35^ ^]^ The modeling approach involved placing a custom‐designed oral device in mice to disrupt their normal dental occlusion and create a UAC. Although this method shares similarities with previous UAC models that primarily examined the abnormal biomechanical loading of the TMJ, our refined protocol produced distinct dentofacial asymmetry outcomes. Through meticulous surgical procedures and comprehensive micro‐CT evaluations, we developed a more precise model that specifically replicates MA characteristics, rather than the broader spectrum of malocclusion effects typically observed in conventional UAC models. This refined approach allows for the targeted investigation of the molecular and structural changes associated with the development of dentofacial asymmetry. In addition, we observed pathological changes in MA mice similar to those observed in UAC mice, such as thinning of the cortical bone, reduction in cartilage thickness, and a decrease in the number of chondrocytes.^[^ ^36^ ^]^ These findings are consistent with the results of imaging and morphological analyses. Unlike conventional UAC models, which primarily focus on TMJ degeneration, our approach precisely captures the structural and biomechanical imbalances unique to MA, thereby enabling more targeted investigations into the neural mechanisms linking malocclusion, chronic pain, and affective disorders.

Previous studies have found that UAC mice exhibit an increase in mechanical allodynia, a decrease in bite force, and a reduction in spontaneous feeding behavior.^[^ ^37^ ^]^ Our findings in the MA model corroborated these observations, demonstrating elevated mechanical hyperalgesia. However, a critical distinction lies in the temporal progression of the pain sensitivity. Although prior studies reported changes at an 8‐week endpoint,^[^ ^38^ ^]^ our MA mice exhibited a significant decrease in pain threshold as early as the second week post‐modeling. Regarding emotional behaviors, a previous study found that mice began to exhibit anxiety‐like behaviors 2 weeks after the establishment of the model, and these behaviors persisted until the sixth week, which is in complete alignment with the findings of our study. However, previous investigations were largely limited to single time‐point assessments (e.g., week 3) and failed to capture the dynamic progression of pain and affective disturbance. Moreover, although prior studies relied on conventional behavioral paradigms (e.g., von Frey testing, elevated plus‐maze, open field),^[^ ^39^ ^]^ which provide valuable but constrained insights, they lacked the analysis of spontaneous behaviors in a more naturalistic setting, an approach that better mirrors the clinical manifestations. To address these limitations, we performed a machine learning‐based ethological analysis to dissect the spontaneous behavioral patterns in MA mice. We observed increasing trends in self‐grooming (a marker of stress and discomfort) and curling‐up postures (indicative of pain‐related guarding behavior), along with reduced exploration and locomotion. Notably, declines in trotting and running behaviors, which require coordinated jaw‐neck movements, suggest functional impairment associated with orofacial discomfort. These fine‐grained behavioral signatures not only reflect spontaneous pain and affective dysfunction in MA mice but also provide a more comprehensive and clinically relevant behavioral profile for this model.

Emerging evidence has shown that craniofacial pain can significantly affect the neuronal excitability and neural plasticity across multiple brain regions, such as the ACC,^[^ ^40^ ^]^ insula,^[^ ^41^ ^]^ and medial TH.^[^ ^42^ ^]^ Through fMOST, linear support vector classification and brain‐wide FOS mapping, we analyzed the brain‐wide activation patterns in the MA mouse model. We discovered that several brain regions, including the ACC, were abnormally activated in MA mice. The ACC is one of the predominant contributors in the discrimination between the CON and MA mice. Previous studies have shown that neurometabolites in the ACC of patients with chronic orofacial pain are significantly altered,^[^ ^43^ ^]^ suggesting a role for the ACC in orofacial neuropathic pain, consistent with our findings. In the present study, integrating data from fMOST, molecular analyses, and electrophysiological recordings of the BLA‐ACC circuit, we propose that transcriptional changes in the ACC are primarily attributed to alterations in synaptic structure and function. Consistent with this proposition, multi‐omics analysis revealed significant perturbations in synaptic function‐related pathways in the MA model. The enriched synaptic function‐associated pathways provide evidence supporting the potential link between transcriptional changes in the ACC and modifications in synaptic structure and function, which warrants further investigation in future studies.

There are an increasing number of studies on the oral–brain axis.^[^ ^44^ ^]^ However, most of these studies were conducted at the level of neuroimaging research, and only involved alterations in a single brain region. The specific neural circuit mechanisms underlying chronic orofacial pain remain unclear. Sensory information transmitted from the oral cavity is relayed to the cerebral cortex via the TH.^[^ ^45^ ^]^ The neural circuits that form between the cerebral cortex and other brain regions after the cerebral cortex receives sensory information remain to be investigated. MA‐induced changes may affect the functional connectivity within the brain network, which could be verified using MRI in future studies. Clinically, patients with chronic orofacial pain often experience pain memories^[^ ^46^ ^]^ and are accompanied by emotional disorders. Therefore, neural circuits related to chronic orofacial pain may exist between the ACC and hippocampus and between the ACC and the amygdala. By performing optogenetics combined with the patch‐clamp recordings in the ACC neurons, we focused on the investigation of the BLA‐ACC pathway in the MA model for its established role in pain‐anxiety comorbidity.^[^ ^31^ ^]^ We provide evidence that the functional synaptic connectivity of the BLA‐ACC circuit was enhanced and presynaptic neurotransmitter release was increased in the MA mice. A more in‐depth exploration of upstream driving factors at the circuit level would lead to a more complete mechanistic framework. Furthermore, investigations targeting other areas except for ACC, such as ORB, SS, RSP and PVH, may develop a more comprehensive circuit‐level understanding of MA‐related pathologies.

Plasticity changes in the ACC play a crucial role in emotional disorders associated with chronic pain.^[^ ^47^, ^48^ ^]^ Specifically, NMDA receptor‐dependent long‐term potentiation (LTP) in the ACC sustains the affective dimensions of chronic pain.^[^ ^49^ ^]^ Blocking presynaptic LTP in the ACC with oxytocin can alleviate the anxiety caused by chronic pain.^[^ ^50^ ^]^ These indicate that plastic changes in the synapses of ACC neurons are of great significance in emotional disorders accompanied by chronic pain. In our MA model, we identified the molecular and structural correlations between these plasticity changes. Western blotting analysis showed upregulation of the synaptophysin and GluR1 subunit, revealing the mediation of synaptic strengthening. Furthermore, the selective upregulation of PKMζ suggests its critical role in sustaining synaptic potentiation and pain‐anxiety comorbidity in chronic orofacial pain, which may be considered as a potential therapeutic target.^[^ ^51^ ^]^ Sparse labeling demonstrated significant dendritic spine remodeling in ACC neurons. These findings align with established chronic pain mechanisms, in which spinal structural plasticity is accompanied by affective disturbances. Electrophysiological recordings provided functional validation, showing enhanced neuronal excitability in MA mice, including increased firing frequency and excitatory synaptic transmission. Our observations are similar to previous findings that have shown that in chronic pain models, when anxiodepressive‐like symptoms occur, the firing frequency and bursting activity of neurons in the ACC increase.^[^ ^52^ ^]^ Notably, our observations bridge multiple scales of analysis, from molecular to structural and functional levels, all converging to suggest ACC hyperexcitability and abnormally enhanced neural plasticity as potential neural substrates for MA‐associated emotional comorbidities.

Our study demonstrated that the chemogenetic inhibition of CaMKII^+^ neurons in the ACC significantly alleviated anxiety‐like behaviors in MA mice, highlighting the critical role of central mechanisms in chronic orofacial pain and emotional comorbidities. Why is central intervention a viable approach for the management of oral diseases? The central nervous system plays a crucial role in pain perception and regulation.^[^ ^53^, ^54^ ^]^ Through central intervention, by modulating the transmission and integration of pain signals, the goal of alleviating pain can be achieved. Moreover, chronic pain is closely associated with negative emotions. We can effectively enhance the emotional status of patients by precisely controlling the brain regions implicated in emotional processing. Previous studies have found that inhibition of pyramidal neurons in the ACC can produce an analgesic effect in chronic inflammatory pain models.^[^ ^55^ ^]^ By shifting the therapeutic paradigm from peripheral symptom management to circuit‐level intervention, our findings provide new possibilities for holistically treating oral diseases, simultaneously mitigating sensory discomfort and psychological distress through mechanisms, such as ACC modulation, which previous studies have shown can induce analgesia in chronic pain models. This approach capitalizes on brain plasticity to achieve durable relief, which is particularly valuable for conditions such as MA, where structural abnormalities trigger maladaptive central sensitization. Our study attempts to bridge oral pathophysiology with systems neuroscience, advocating for integrated treatment strategies that address both the local etiology of dental disorders and their far‐reaching neural consequences, ultimately redefining therapeutic success in terms of both biological and psychological recovery.

Imaging evidence has previously demonstrated that the ACC is persistently activated in patients with chronic orofacial pain,^[^ ^41^ ^]^ with increased functional connectivity to other brain regions such as the left anterior insula.^[^ ^56^ ^]^ ACC holds significant translational potential for targeted intervention in MA, yet it is accompanied by numerous challenges that warrant evaluation. Clinical studies have confirmed that repetitive transcranial stimulation targeting ACC exerts anxiolytic effects in patients with central neuropathic pain.^[^ ^57^ ^]^ Its application in the intervention of MA holds substantial promise, as it may disrupt the vicious cycle between pain and negative emotions. However, limitations persist, for example, the anatomical depth of the ACC and inter‐individual variations in its location may compromise stimulation precision, and optimal stimulation parameters (such as frequency, intensity, and duration) remain to be explored. Pharmacological strategies offer an alternative approach, with potential targets including the glutamatergic,^[^ ^58^ ^]^ serotonergic,^[^ ^59^ ^]^ and endocannabinoid^[^ ^60^ ^]^ systems within the ACC—all of which are involved in synaptic plasticity and nociceptive signaling. For instance, drugs targeting NMDA receptors or AMPA receptors can alleviate ACC hyperactivation by regulating excitatory synaptic transmission; selective serotonin reuptake inhibitors (SSRIs) may modulate the affective component of pain mediated by the ACC through serotonergic projections. However, pharmacological approaches have inherent limitations: systemic administration often lacks regional specificity, increasing the risk of cognitive or affective side effects.

Furthermore, targeted interventions on the ACC are still constrained by a therapeutic window, which includes both temporal and parameter‐based dimensions. Clinical interventions should be initiated before pain chronification, during which neural plasticity remains unfixed. For instance, early application of transcranial magnetic stimulation (TMS) targeting the ACC can inhibit the reciprocal amplification of pain and anxiety; if delayed to the chronic phase, higher stimulation intensities may be required. Regarding the parameter window, for repetitive transcranial magnetic stimulation (rTMS) targeting the ACC, attention must be paid to stimulation intensity and single‐session duration. In terms of pharmacotherapy, emphasis should be placed on the effective dosage of drugs and associated side effects.

In conclusion, this study establishes mandibular asymmetry as a preclinical model of TMD‐related chronic pain and anxiety, revealing a critical role for ACC hyperactivation and synaptic plasticity in mediating these comorbidities. Longitudinal behavioral and multi‐scale omics analyses demonstrated that structural craniofacial defects drive maladaptive neural reorganization, with ACC CaMKII^+^ neurons exhibiting heightened excitability, altered transcriptional profiles, and enhanced synaptic plasticity. Functional interventions confirmed that modulating ACC activity alleviates both pain and anxiety‐like behaviors, highlighting its crucial role in pain‐anxiety comorbidity. These findings provide a mechanistic link between maxillofacial anatomical abnormalities and neurobehavioral dysfunction, positioning the ACC as a key target for neuromodulatory therapies in TMD and related orofacial disorders.

## 4 Experimental Section

### Animals

Male and female adult C57BL/6J mice (20‐25 g) were obtained from the Fourth Military Medical University animal facility, and were housed in a temperature‐controlled environment (22‐25 °C) under a 12‐h light/12‐h dark cycle. The animals were randomly assigned to different experimental groups. All procedures were approved by the Institutional Animal Care and Use Committee of the Fourth Military Medical University (FMMULL‐20221120) and conformed to the Guide for the Care and Use of Laboratory Animals published by the National Institutes of Health (NIH). Animals were randomly allocated to experimental groups using a computer‐generated randomization sequence prepared prior to study initiation.

### Mandibular Asymmetry (MA) Model

The mice were anesthetized with isoflurane (4% for induction and 1.5% for maintenance). Subsequently, the defective restorations were bonded to the left maxillary and mandibular incisors of the mice to induce artificial occlusal interference, compelling the mice to exhibit adaptive mandibular displacement, thus simulating MA. To create a defective maxillary restoration, a disposable sterile injection needle (1.2 mm) was cut into a 1‐mm metal tube. One end of the injection needle was flattened into a 1‐mm guide plate with pliers and bent at a 45° angle, while ensuring that the length of the short tube was 2 mm. After restoration, moisture isolation was performed, and the restoration was bonded to the left maxillary and mandibular incisors of the mice with dental zinc phosphate cement. Regular inspections were performed during the experiment to ensure that the restorations were well fixed. The mice in the CON group were not subjected to any treatment, and all the animals were housed under the same housing conditions.

### Infraorbital Nerve Chronic Constriction Injury (IoN‐CCI) Model

The mice were anesthetized with isoflurane (4% for induction and 1.5% for maintenance), and the facial surface between the eye and the whisker pad was gently shaved without damaging the whiskers. A 0.5 cm incision was made parallel to the ipsilateral orbit, starting caudal to the third row of whiskers. After blunt dissection of the superficial fascia, the main trunk of the infraorbital nerve was exposed at its distal segment. Two ligatures (6‐0 suture) were loosely tied around the distal portion of the IoN with an interval of approximately 1 mm. To ensure adequate constriction, the ligatures were tightened until a slight reduction in nerve diameter and transient slowing of superficial blood flow were observed, while preserving overall vascular patency and without transecting the nerve. Finally, the skin incision was sutured closed, and the mice were placed into their cages.^[^ ^61^ ^]^

### Micro‐Computed Tomography

Micro‐CT imaging (Quantum GX2, PerkinElmer) was used to observe the TMJs of the mice in the CON and MA groups. The region of interest for scanning was the entire skull. The establishment of the mouse model was evaluated using micro‐CT images and their 3D reconstructions. BV fraction and TB.Th were measured to evaluate the successful establishment of the MA model.

### Pain Behavioral Test

The mice were placed in the behavioral room for 1 h before the behavioral test to acclimatize them to the environment, and a specially made metal net cage (4 × 4 × 10 cm) was used to detect pain‐related behavior. After the mice were in a quiet state, von Frey filaments (with specification values of 1.65, 2.36, 2.44, 2.83, 3.22, and 3.61) were used to poke the masseter muscle area or the whisker pad of the left maxillofacial region within 1 cm above and below the midpoint of the line connecting the outer canthus of the eye and the external auditory meatus. The same specification of the filament was tested ten times, and the corresponding value of the smallest specification of the filament that caused the mice to show positive behaviors more than six times was recorded as the pain threshold. During detection, when the animal showed rapid retraction of its head or continuous face‐rubbing behavior several times, it was considered a positive response to the stimulus. The minimum inter‐stimulus interval for the von Frey test was 5 min.

### Open Field Test

The open field test was conducted in a box with dimensions of 50 cm × 50 cm × 40 cm. At the start of the experiment, the mice were placed in the central zone of the box. The activity of the mice was recorded using a video recording for 10 min. The test box was thoroughly cleaned with 70% ethanol to remove any residual odor. The measured parameters included the number of times each mouse entered the central area, time spent in the central area, percentage of moving distance in the central area, and total moving distance. Analysis was performed using SMART software (version 3.0; Panlab Harvard 11 Apparatus, Newbury Park, CA, USA).

### Elevated Plus‐Maze Test

The elevated plus‐maze test was performed in a maze composed of two open arms (30 × 5 cm) and two closed arms (30 × 5 × 30 cm). The open and closed arms extended outward at a 90° angle from the central platform, with dimensions of 5 cm × 5 cm. At the start of the test, the mice were placed on a central platform facing an open arm. The activity of the mice was recorded using a video recording for 10 min. The maze was thoroughly cleaned with 70% ethanol to remove any residual odor. The measured parameters included the number of times the mouse entered the open arm area, time spent in the open arm area, percentage of moving distances in the open arm area, and total moving distance. The analysis was performed using the SMART software (version 3.0; Panlab Harvard 11 Apparatus, Newbury Park, CA, USA).

### 3D Fine Behavior

The mice were placed in the behavioral room 30 min before the behavioral test to acclimatize them to the environment. Each mouse was allowed to move freely in a transparent circular open field with a diameter of 50 cm. A camera (Intel RealSense D435) was placed on each of the four sides of the device to record the behavior of the mice synchronously for 10 min. Synchronous videos were obtained using multi‐view cameras in a 3D motion capture system. Subsequently, the Behavior Atlas was used to automatically identify the behavioral phenotypes of the mice.^[^ ^62^ ^]^

### Facial Parameters

It has been well established that a range of facial parameters serve as robust indicators of the pain state in mice.^[^ ^63^ ^]^ Based on these findings, several easily visualized landmarks were identified on lateral‐view images of mice to enable precise quantification of changes in the face. Multiple reference points were tested to capture changes related to the mouth, nose, and ears, including measurements of the visible area of the inner ear. The final set of reference points for each facial parameter was selected to maximize sensitivity to facial changes while minimizing parameter redundancy. Following extensive evaluation, seven facial parameters were captured that quantitatively captured relevant changes in facial displays: eye opening, ear opening, ear angle, ear position, mouth position, snout position, and face inclination. To avoid confounding effects from camera zoom or individual size differences, all retained parameters were expressed as ratios or angles.

### Fluorescence Micro‐Optical Sectioning Tomography

The AAV vector rAAV_PHP.eB_‐cFos‐EYFP was purchased from Brain Case Biotechnology Co., Ltd. The viral stocks had titers of 3.89 × 10^12^ viral genomes/mL and were stored at –80 °C. Mice were administered 2 × 10^11^ viral genomes/mouse in 200 µL sterile phosphate‐buffered saline (PBS) via the lateral caudal vein. They were anesthetized with an overdose of 2% pentobarbital sodium and perfused transcardially with PBS, followed by 4% paraformaldehyde (PFA) (Millipore Sigma, Billerica, MA, USA) at 4 °C for 24 h. A solution was prepared using a mixture of agarose powder (Sigma‐Aldrich) and distilled water at concentrations of 3–5%. Post‐fixation mouse brains were embedded in an agarose solution, and the position was adjusted to ensure that the main axis of the mouse brain was parallel to the edge of the sample container. The sample was allowed to rest for 8–10 min until it solidified. The solid blocks were then removed and fixed onto the sample holder using glue. The light sheet imaging system adopted a tomographic imaging strategy, and the thickness of each image was 46 µm. Image preprocessing for mouse brain data set at the voxel resolution of 1.20 × 1.20 × 0.92 µm^3^ was executed on a computing server (32 cores, 2.9 GHz per core).

Then, by using the fitcsvm package in MATLAB software (version R2022a, Mathworks, USA), the linear support vector classification was performed to identify MA‐induced activation patterns from a whole brain perspective. The total number of FOS‐EYFP^+^ cells in each area was standardized into z‐score data. The classifier weights in each classification were transformed into corresponding activation patterns using the following formula:^[^ ^28^, ^29^ ^]^

(1) $$\hat{s}\left(n\right)=W^{T}x\left(n\right)$$

(2) $$A=\Sigma_{x}W\Sigma_{\hat{s}}^{-1}$$

(3) $$x\left(n\right)=\mathrm{As}\left(n\right)+\varepsilon \left(n\right)$$

where $\Sigma_{\hat{s}}=W^{T}\Sigma_{x}W$

A is the M × K activation pattern matrix in the forward model, where M denotes the number of brain areas and K denotes the number of target variables in each area of the group. The vector x represents the M‐dimensional observed data—in this case, z‐scores across 129 areas (M = 129). The vector $\hat{s}$ corresponds to the K‐dimensional latent factors. Additionally, Σ_x_ denotes the data covariance matrix, W is the M × K weight matrix from the backward model (i.e., the classifier weights derived from linear support vector machine classification), and $\Sigma_{\hat{s}}$ represents the covariance matrix of the latent factors.

### Immunofluorescence

After anesthetizing the mice with 2% pentobarbital sodium, a cannula was inserted into the ascending aorta through the left ventricle to ensure complete perfusion of the circulatory system. First, the blood was washed with PBS (0.01 M, pH 7.4), and the animals were perfused with 4% PFA (Millipore Sigma, Billerica, MA, USA). The brains of the mice were removed, fixed in 4% PFA for 6 h, and then immersed in a 30% sucrose solution for dehydration. After the brain was completely immersed in the sucrose solution, a cryostat microtome (Leica CM 1950; Leica Microsystems, Wetzlar, Germany) was used to prepare sections with a thickness of 30 µm. First, the sections were rinsed in PBS solution (thrice, 10 min each time) and incubated with primary antibodies (rabbit anti‐FOS: 1:1500, Cat#: 226 008, Synaptic Systems; mouse anti‐CaMKII 1:500, Cat#: ab52476, Abcam) at 4 °C for 24 h. Then, the sections were rinsed again in the same way and incubated with the appropriate secondary antibodies (Donkey anti‐rabbit IgG (H+L) Alexa Fluor 594 1:500 Cat#: A21207, Invitrogen; Donkey Anti‐Guinea Pig IgG (H+L) Alexa Fluor 647 1:500 Cat#: 705 006 148, Jackson Laboratories; Donkey anti‐rabbit IgG(H+L) Alexa Fluor 488 1:500 Cat#: A21206, Invitrogen; Donkey anti‐rabbit IgG(H+L) Alexa Fluor 647 1:500 Cat#: 711‐605‐152, Jackson Laboratories) at room temperature for 3 h. Finally, the sections were air‐dried, mounted with a mixture of 50% (v/v) glycerol and 2.5% (w/v) triethylenediamine dissolved in 0.01 M PBS, and covered with a cover slip. Panoramic images were obtained using a VS200 microscope (10×; Olympus, Tokyo, Japan), and confocal images of the brain sections were obtained using an Olympus FV3000 microscope (20×). To determine the brain regions activated during pain induced by TMJ osteoarthritis, FOS immunofluorescence staining was performed on each brain section.

### Stereotaxic Surgery

The mice were deeply anesthetized with isoflurane (4% for induction and 1.5% for maintenance). The anesthetized mice were fixed on a stereotaxic apparatus (RWD Life Science Inc., Shenzhen, China). After shaving and disinfecting the head, the scalp was cut open with scissors. The bregma point was set as zero, and the left and right sides were leveled so that when the X‐axis was ± 2.00 mm, the difference between the Z‐axis values on the left and right sides was < 0.03 mm. A hole was drilled in the skull of the mouse for virus injection, and a microsyringe needle (10 µL) was used to inject the virus (180 nL, 30 nL/min). The injection coordinates for the ACC were as follows: the anterior–posterior (AP) coordinate was 0.82 mm, the medial–lateral (ML) coordinate was 0.30 mm, and the dorsal–ventral (DV) coordinate was –1.75 mm. For the fiber photometry experiment, a fiber optic (200 µm OD, 0.5 NA, NewDoon, China) was implanted after the virus injection (AP coordinate, 0.82; ML coordinate, 0.30; DV coordinate, –1.72) and fixed with 3 M flowable resin.

### In Vivo Fiber Photometry Recording

One week after injecting the rAAV‐CaMKII‐GCaMp6s‐WPRE‐hPA virus and implanting the fiber optics, the model was established. The CON group received the same virus injection and fiber optic implantation, but did not undergo the modeling operation. Two weeks after modeling, a device (FPS‐410/470; Inper Ltd., China) was used to record the data. During the experiment, a 470‐nm laser was used to detect the calcium‐dependent GCaMP6s fluorescence emission signal, and a 410‐nm laser was used to detect the isosbestic point control signal. When the mouse was in a quiet and stable state, a von Frey filament with a value of 3.22 was used to gently poke the masseter muscle area of the left maxillofacial region of the mouse within a range of 1 cm above and below the midpoint of the line connecting the outer canthus of the eye and the external auditory meatus. After collection, the Inper Data Process software (Inper Studio) was used for data processing to analyze the calcium transient change value (ΔF/F). To ensure the accuracy of the experiment, all mice were perfused after the experiment was completed to determine whether the virus injection site and fiber optic implantation position met the experimental expectations.

### Integrating Analysis of Single‐Cell RNA Sequencing and Spatial Transcriptomics

Single‐nucleus RNA sequencing (snRNA‐seq) and spatial transcriptomics were integrated to characterize cellular heterogeneity and spatial gene expression patterns.^[^ ^64^ ^]^ For snRNA‐seq, ACC was rapidly microdissected from fresh‐frozen brains, and nuclei were extracted using a detergent‐based lysis buffer followed by purification via density gradient centrifugation. Libraries were constructed with the 10× Genomics Chromium Single Cell Reagent Kit and sequenced on an Illumina NovaSeq 6000 platform. For spatial transcriptomics, 10‐µm‐thick coronal cryosections containing the ACC were mounted onto visium spatial gene expression slides. Sections were stained with H&E for histological annotation, imaged at high resolution, and then permeabilized to enable mRNA capture by spatially barcoded oligonucleotides (Genergy Biotechnology Co., Ltd.). After cDNA synthesis and amplification, libraries were prepared and sequenced. snRNA‐seq data were processed using the Cell Ranger pipeline for alignment, barcode counting, and UMI aggregation. Downstream analyses, including normalization, PCA, UMAP projection, and graph‐based clustering, were conducted in Seurat. Cell types were annotated using canonical markers. Spatial transcriptomics data were processed with Space Ranger to align transcripts to histological spots. Integration of snRNA‐seq and spatial data was performed using robust cell type decomposition and non‐negative matrix factorization based methods to infer cell type localization and assess spatially restricted gene expression programs within the ACC.

### Chemogenetics

The mice were divided into three groups, namely, the EYFP, Gq, and Gi groups, and appropriate viruses were injected bilaterally: rAAV‐CaMKII‐EGFP‐WPRE‐hGHPA, rAAV‐CaMKII‐hM3Dq(Gq)‐mCherry‐WPRE‐hGHPA, and rAAV‐CaMKII‐hM4Di (Gi)‐EGFP‐WPRE‐hGHpA. One week after the virus injection, the model was established, and behavioral tests were performed 2 weeks after modeling. Forty minutes before the behavioral test, the mice were injected with 100 µL of CNO (0.75 mg/kg; the solvent used was dimethyl sulfoxide (DMSO). To ensure the accuracy of the experiment, all mice were perfused after the experiment was completed to check whether the virus injection site met the experimental expectations.

### Sparse Labeling

The mice were anesthetized with isoflurane (4% for induction and 1.5% for maintenance). A mixture of 100 µL of AAV_PHP.eB_‐hSyn1‐EGFP‐P2A‐EGFP (PackGene Biotech Inc., Guangzhou, China) with a titer of 1.3 × 10^12^ gc/mL was injected into the retro‐orbital venous sinus with a 0.5 mL syringe equipped with a 29 G needle. The mice were housed in a breeding environment. The MA group was modeled 2 weeks after virus injection, and perfusion sections were prepared 4 weeks after virus injection, with a section thickness of 300 µm.

### Western Blotting

Brain tissue (ACC) was homogenized in radioimmunoprecipitation assay lysis buffer (0.5% NP‐40, 150 mM NaCl, 1 mM EDTA, 10 mM Tris, and 1% Triton X‐100 at pH 7.4) containing a protease (Cat# 43 002 700, Roche) and phosphatase inhibitor (Cat# 41 659 200, Roche). Protein concentration was determined using a BCA protein quantification kit (Cat# 23 225, Thermo Scientific). After separation by 10% or 12% sodium dodecyl sulfate‐polyacrylamide gel electrophoresis, the proteins were transferred to a 0.22‐micron polyvinylidene fluoride membrane (Cat#: 0 301 004 0001, Roche Diagnostics Gmbh). The membrane was blocked with tris‐buffered saline containing 5% skim milk and 0.1% tween 20. Then, it was incubated with the primary antibodies overnight at 4 °C as follows: rabbit anti‐synaptophysin (1:20 000, Abcam# 32 127), rabbit anti‐NR2B (1:1000, Abcam# 65 783), and rabbit anti‐glutamate receptor 1 (1:2000, Abcam#183 797), rabbit anti‐Homer1 (1:1000, Abcam#184 955), rabbit anti‐PSD95 (1:2000, Abcam#18 258), rabbit anti‐PKMζ (1:2000, Proteintech#26899‐1‐AP), rabbit anti‐PRKAR2B (1:1000, Proteintech#26899‐1‐AP), rabbit‐anti‐CaMKII (1:2000, Abcam#ab534756), mouse anti‐β‐actin (1:20 000, Proteintech#66009‐1‐Ig) and rabbit anti‐β‐tubulin (1:2000, Proteintech#10094‐1‐AP). After washing with tris‐buffered saline containing 0.1% Tween 20, the membrane was incubated with a horseradish peroxidase‐labeled anti‐rabbit antibody (1:5000, Proteintech #SA00001‐2) or anti‐mouse antibody (1:5000, Proteintech #SA00001‐1) at room temperature for 2 h. An enhanced chemiluminescence reagent kit (Cat# 32 106; Thermo Fisher Scientific) was used to **visualize the protein bands**. Gray values were quantitatively analyzed using ImageJ software to determine the expression levels of target proteins relative to β‐actin.

### Electrophysiological Recording

Whole‐cell patch‐clamp recordings were conducted as previously outlined.^[^ ^65^ ^]^ Mice were anesthetized using isoflurane, followed by transcardial perfusion with ice‐cold, carbogenated (95% O_2_, 5% CO_2_) cutting solution composed of: 115 mM choline chloride, 2.5 mM KCl, 1.25 mM NaH_2_PO_4_, 0.5 mM CaCl_2_, 8 mM MgCl_2_, 26 mM NaHCO_3_, 10 mM D‐(+)‐glucose, 0.1 mM l‐ascorbic acid and 0.4 mM sodium pyruvate (300–305 mOsml^−1^). Coronal brain slices (300 µm thick) encompassing the ACC (corresponding to Bregma 1.42 to 0.50 mm, localized based on the morphology of the lateral ventricles and corpus callosum) were prepared with a vibratome (VT1200S, Leica, Germany). Whole‐cell patch‐clamp recordings were performed at 28–30 °C under infrared differential interference contrast optics, with perfusion of artificial cerebrospinal fluid (ACSF) containing: 119 mM NaCl, 2.3 mM KCl, 1.0 mM NaH_2_PO_4_, 26 mM NaHCO_3_, 11 mM D‐(+)‐glucose, 1.3 mM MgSO_4_, and 2.5 mM CaCl_2_ (pH 7.4, 295–300 mOsml/L). Pyramidal neurons in the ACC were identified based on their morphological and electrophysiological characteristics using differential interference contrast microscopy. Recordings were acquired with a Multiclamp 700B amplifier (Molecular Devices), filtered at 5 kHz, and sampled at 20 kHz via a Digidata 1550B. Data acquisition and analysis were carried out using Clampex 10.7 software.

For paired‐pulse ratio (PPR) measurements, the cell membrane potential was clamped at −60 mV, and excitatory postsynaptic currents (EPSCs) were triggered via a 2‐ms light stimulation at 470 nm. Paired‐pulse stimuli were delivered with interstimulus intervals of 50, 100, and 200 ms. The PPR was determined by dividing the peak current elicited by the second stimulus by the peak current evoked by the first stimulus.

### Drug Administration

For analgesia studies, gabapentin (G122413, Aladdin) was administered intraperitoneally at a dosage of 100 mg/kg body weight. This pharmacological intervention commenced one week following the establishment of the MA model, and was administered once daily for seven consecutive days. Mice in the corresponding control group received equivalent volumes of saline on the same schedule. All injections were performed at a consistent time each day to minimize circadian influences on behavioral testing.^[^ ^66^ ^]^

### Statistical Analyses

Statistical analyses were performed using GraphPad Prism (version 10.1.2). All data were expressed as mean ± standard error of the mean. The raw data and details of statistical tests were provided in Table S1 (Supporting Information). Briefly, the Shapiro‐Wilk test and Levene's test were used to assess normality and homogeneity of variances, respectively. For data meeting both assumptions, two‐tailed unpaired t‐test, one‐way analysis of variance (ANOVA) with Bonferroni correction for post‐hoc testing, and repeated‐measures ANOVA with simple effects analysis were applied. Non‐normally distributed datasets were analyzed using non‐parametric tests. P < 0.05 was considered statistically significant.

## Conflict of Interest

The authors declare no conflict of interest.

## Supporting information



Supporting Information

Supplemental Table 1

Supporting Information

Supplemental Video 1

Supplemental Video 2

Supplemental Video 3

## Data Availability

The data that support the findings of this study are available from the corresponding author upon reasonable request.
