# Supplementary material for: Synaptic Dysfunction in the Anterior Cingulate Cortex Underlies Pain‐Anxiety Comorbidity in a Mandibular Asymmetry Mouse Model
Source: Adv Sci (Weinh). 2025 Sep 24;12(46):e09509. doi: 10.1002/advs.202509509 (PMC12697876; doi:10.1002/advs.202509509)
Supplement: Supplementary file 1 — Supporting Information [file ADVS-12-e09509-s001.docx]

Supporting Information

Synaptic Dysfunction in the Anterior Cingulate Cortex underlies Pain-Anxiety Comorbidity in a Mandibular Asymmetry Mouse Model

Zhaoyichun Zhang^1,2†^, Yanran Zhang^2†^, Jialin Si^3^, Honghui Mao^2^, Feng He^4^, Jin Ning^5^, Guaiguai Ma^2^, Xiaohua Chen^4^, Haoxiang Xiao^2^, Yuanyuan Zhu^2^, Haifeng Zhang^2^, Yifan Lu^2^, Qian Liu^2^, Meng Nian^3^, Shiquan Sun^5^, Shibin Yu^4^, Shengxi Wu^2^, Ze Fan^2,6*^, Zuolin Jin^1*^, Jing Huang^2*^

Supporting Information


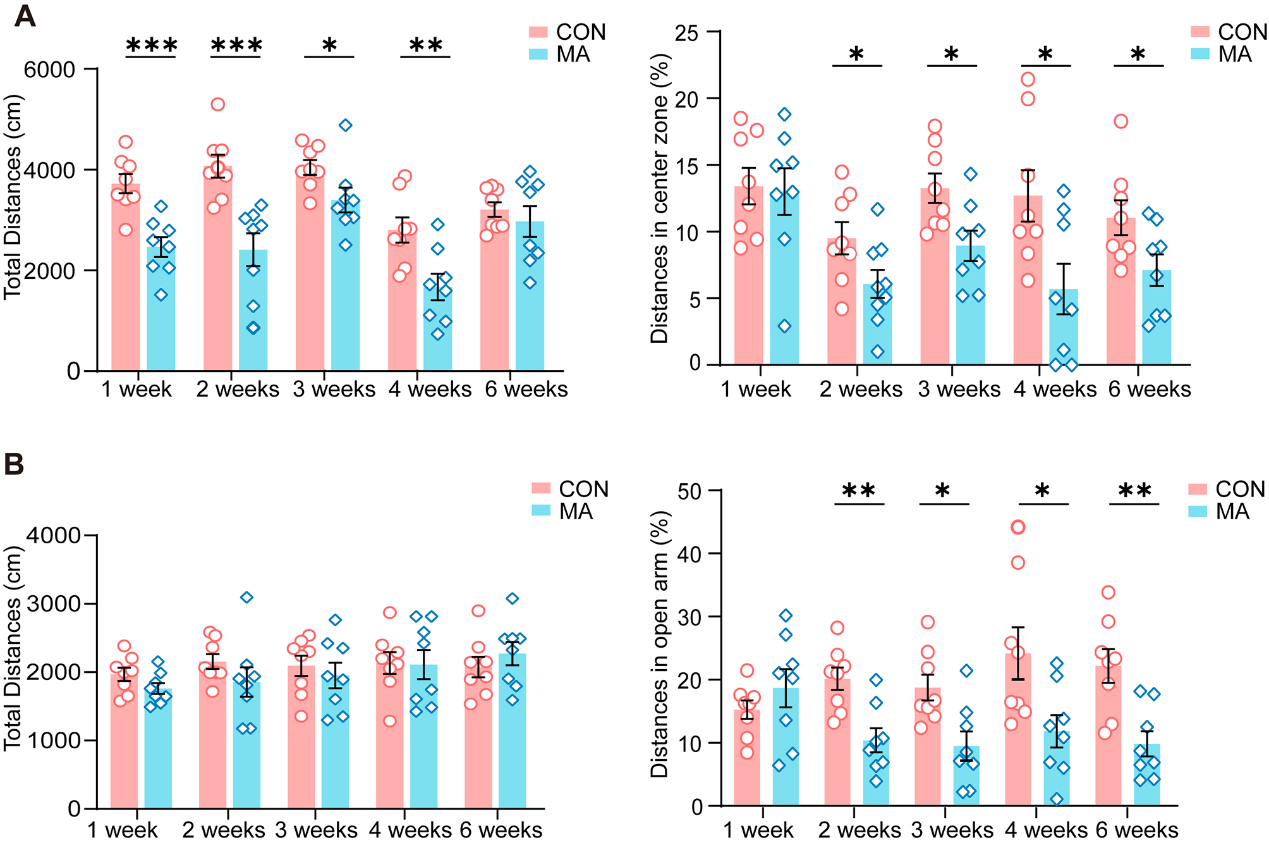


**Fig. S1 Comparison of the total distances of mice in CON and MA groups in the open field and elevated plus-maze tests**

A) In the open field test total distances traveled and distances in the center zone are shown across 1 - 6 weeks. B) In the elevated plus-maze, total distances and those in the open arm are presented over the same time span. Statistical significance indicates distinct anxiety-like phenotypes in the MA group relative to CON (n = 8). *P < 0.05, **P < 0.01, ***P < 0.001, by Two-tailed unpaired separate variance estimation t-test and Two-tailed unpaired t test. Data are presented as the mean ± SEM.


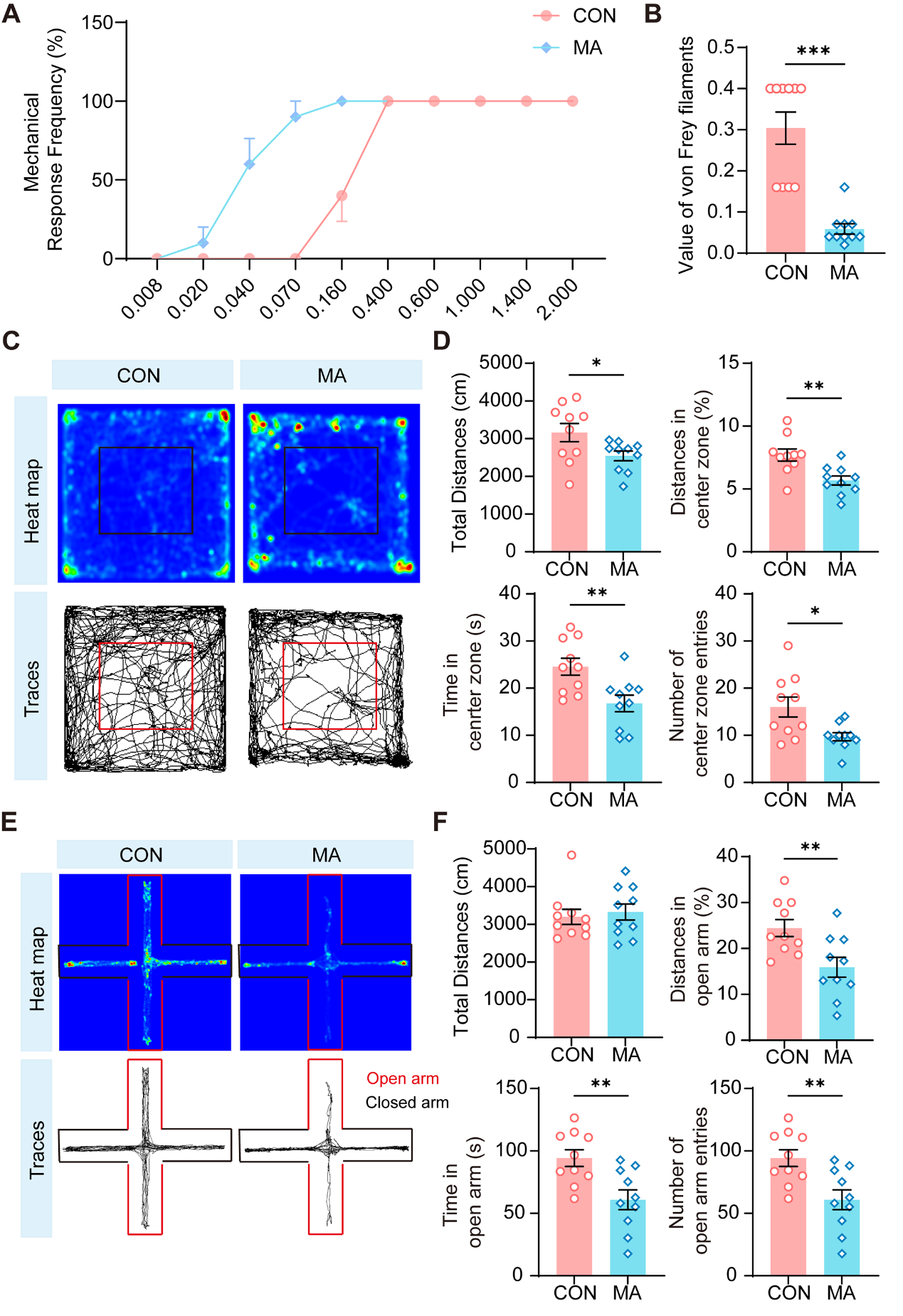


**Fig.S2 Pain and anxiety-related behaviors in female MA model mice**

A) Mechanical response frequency to von Frey filaments of different forces in CON and MA mice. B) Quantification of the von Frey filament value (n = 10). C) Heat maps and movement traces in the open field test. D) Quantification of total distances, distances in the center zone, time in the center zone, and number of center zone entries (n = 10). E) Heat maps and traces in the elevated plus maze test. F) Quantification of total distances, distance in open arms, time in open arms, and number of open arm entries (n = 10). *P < 0.05, **P < 0.01, ****P < 0.0001, by two-tailed unpaired t test Mann-Whitney U test. Data are presented as mean ± SEM.


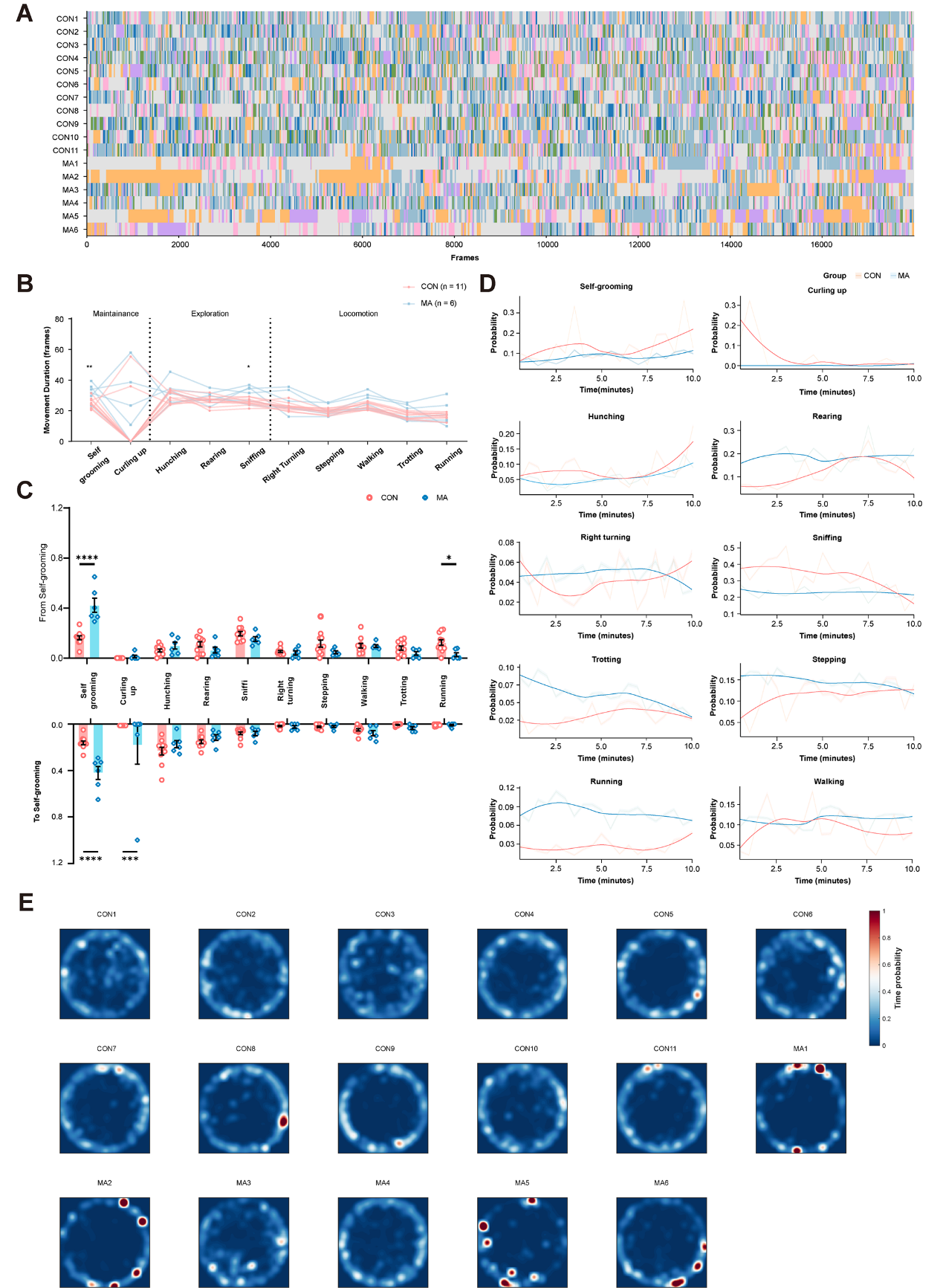


**Fig. S3 Analysis of the differences in the time, probability, and proportion of spontaneous behaviors between the CON and MA group**

1. Ethograms of 11 samples in the CON group and 6 samples in the MA group. B) Results of differential analysis of the average duration of each action within the group (n = 11 (CON), n = 6 (MA)). C) (Top) Results of differential analysis of the probabilities of the face-rubbing behavior transitioning to other actions; (Bottom) Results of differential analysis of the probabilities of other behaviors transitioning to the face-rubbing behavior (n = 11 (CON), n = 6 (MA)). D) Line graphs showing the changes in the action proportion of each action in 30-second intervals. E) Location heat maps of each sample. *P < 0.05, **P < 0.01, by Two-way ANOVA, Two-tailed unpaired t test, Mann-Whitney U test, and Two-tailed unpaired separate variance estimation t-test. Data are presented as mean ± SEM.


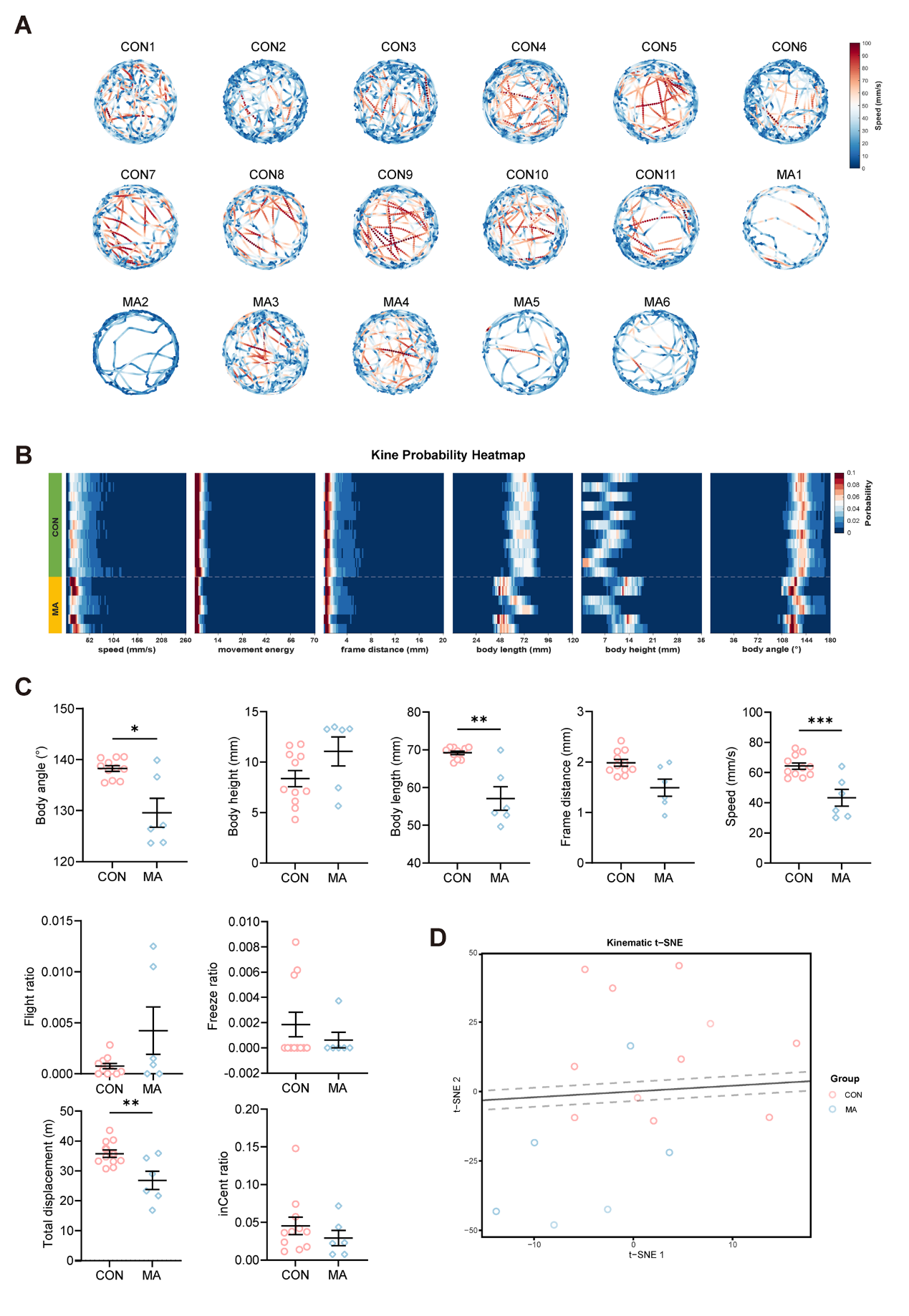


**Fig. S4 Characterization and comparison of kinematic parameters of spontaneous movements between the CON and MA group mice: visualization, statistical analysis, and classification results**

A) Heatmaps of the velocity trajectories for each sample. B) Frequency heat maps of the movement speed, movement intensity, displacement distance per frame, body length, body height, and body angle of the mice each row represents all frame data of one sample. C) Results of the differential analysis of kinematic parameters. The body angle, body length, displacement distance per frame, and movement speed of the CON group are significantly higher than those of the MA group (n = 11 (CON), n = 6 (MA)). D) t-SNE dimensionality reduction results of 39 kinematic parameters and the decision results of the SVM classifier. *P < 0.05, by Two-tailed unpaired separate variance estimation t-test, Mann-Whitney U test. Data are presented as mean ± SEM.


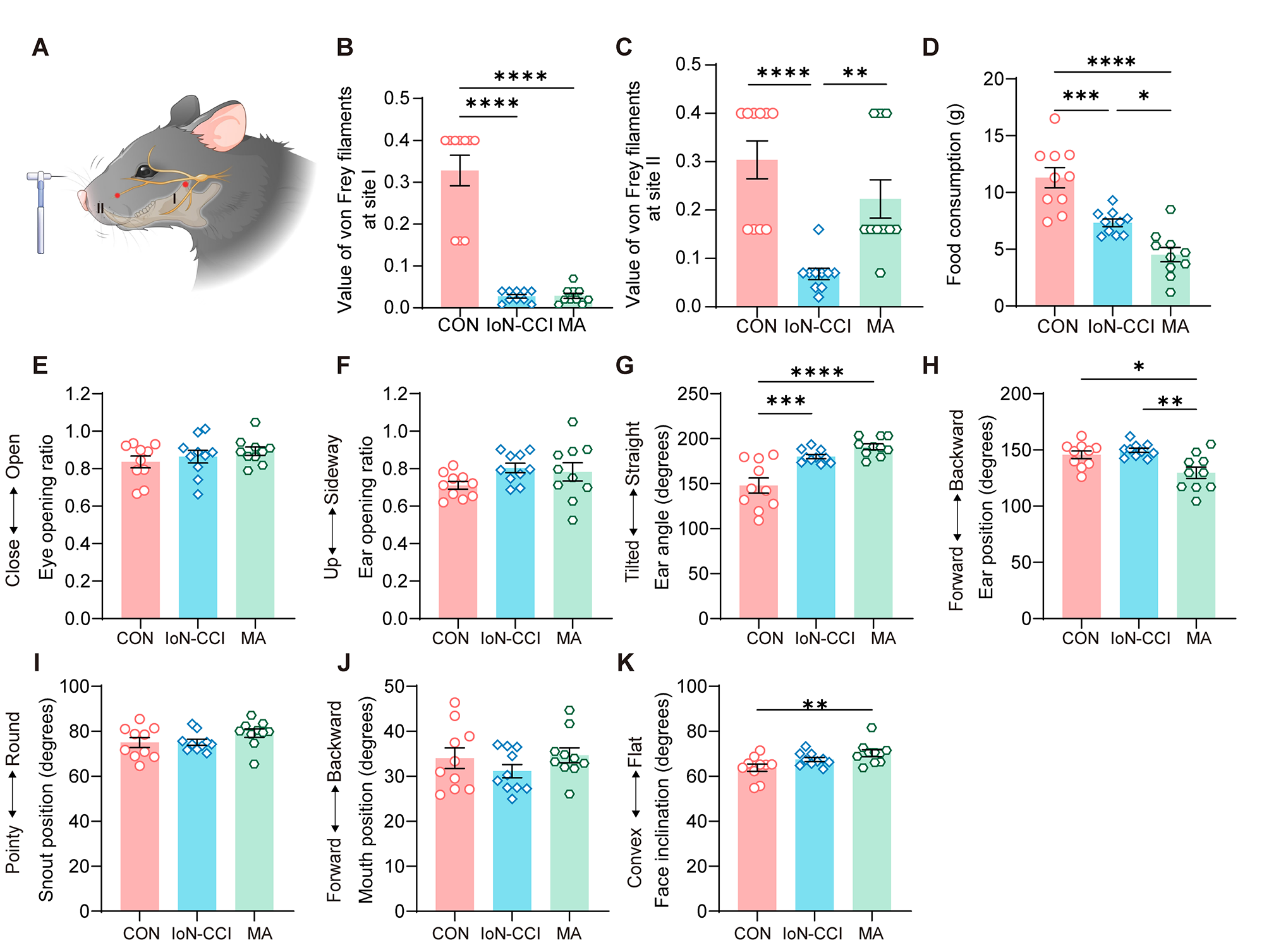


**Fig. S5 Comparison of pain threshold, food intake, and facial expressions in different regions between MA and IoN-CCI models**

A) Systematic description of the testing sites on the mouse face, with a dental hammer indicating the stimulation area relevant to the MA and IoN-CCI models. B-C) Quantification of the pain thresholds at different orofacial points (site Ⅰ and site Ⅱ) using von Frey filaments. D) Food consumption data show that MA mice exhibit significantly lower food intake than both the IoN-CCI model and CON group. E-K) Analysis of multiple facial expression parameters, including eye opening ratio (E), ear opening ratio (F), ear angle (G), ear position in different directions (H), snout position (I), mouth position (J), and face inclination (K). *p < 0.05, **p < 0.01, ***p < 0.001, ****p < 0.0001, by Kruskal-Wallis H test, One-way ANOVA, Welch ANOVA, *post hoc* Dunn multiple comparison, Tukey multiple comparison Dunnett, T multiple comparison. Data are presented as mean ± SEM.


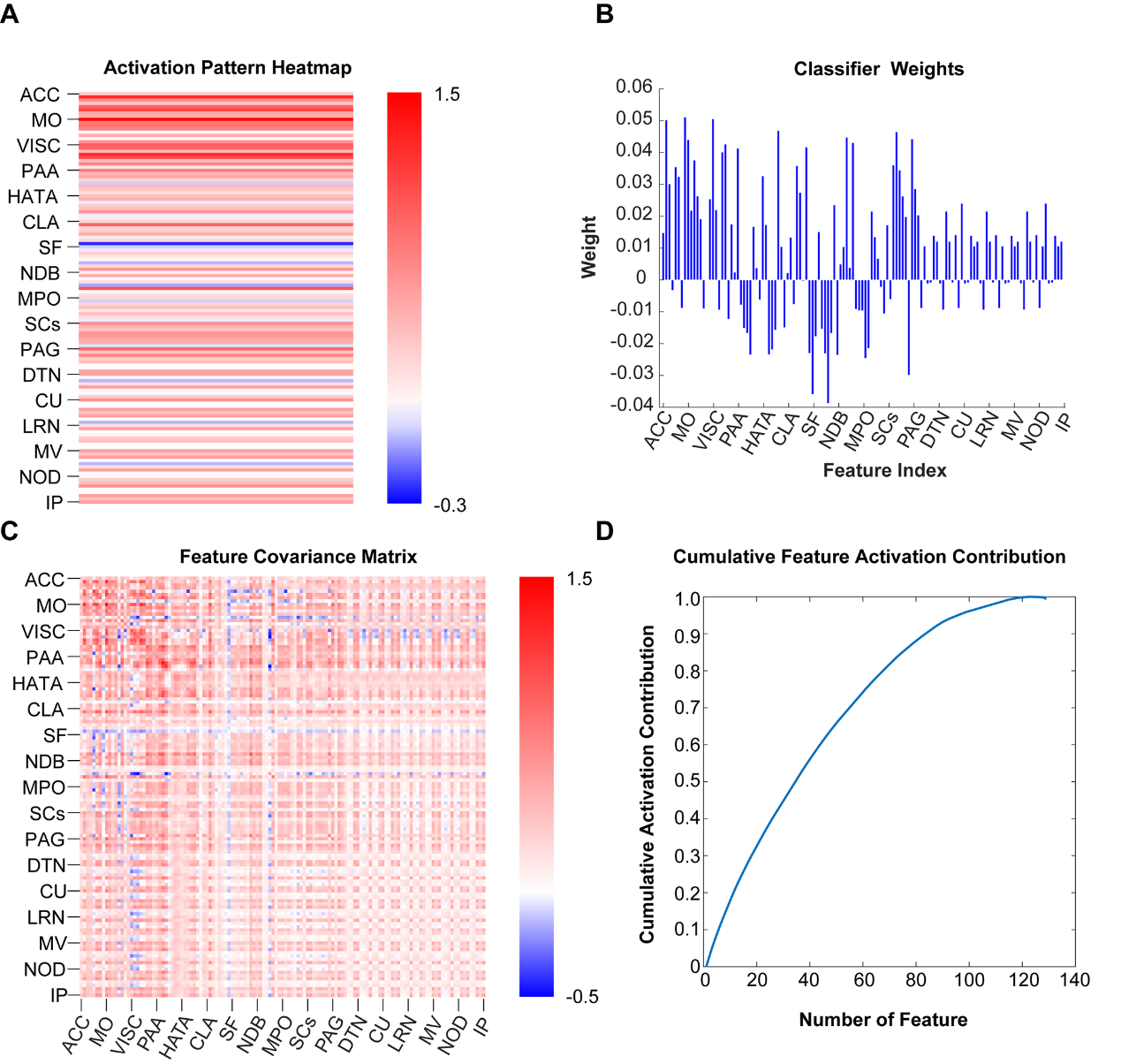


**Fig. S6 The Activation pattern of brain regions in MA mouse model**

A) The activation pattern heatmap shows that the ACC is one of the predominant contributors in the discrimination between the CON and MA mice. B) The classifier weight of each brain region in the activation pattern of MA mouse model. C) The feature covariance matrix of the activation pattern. D) The curve of the cumulative feature activation contribution in MA mouse model.


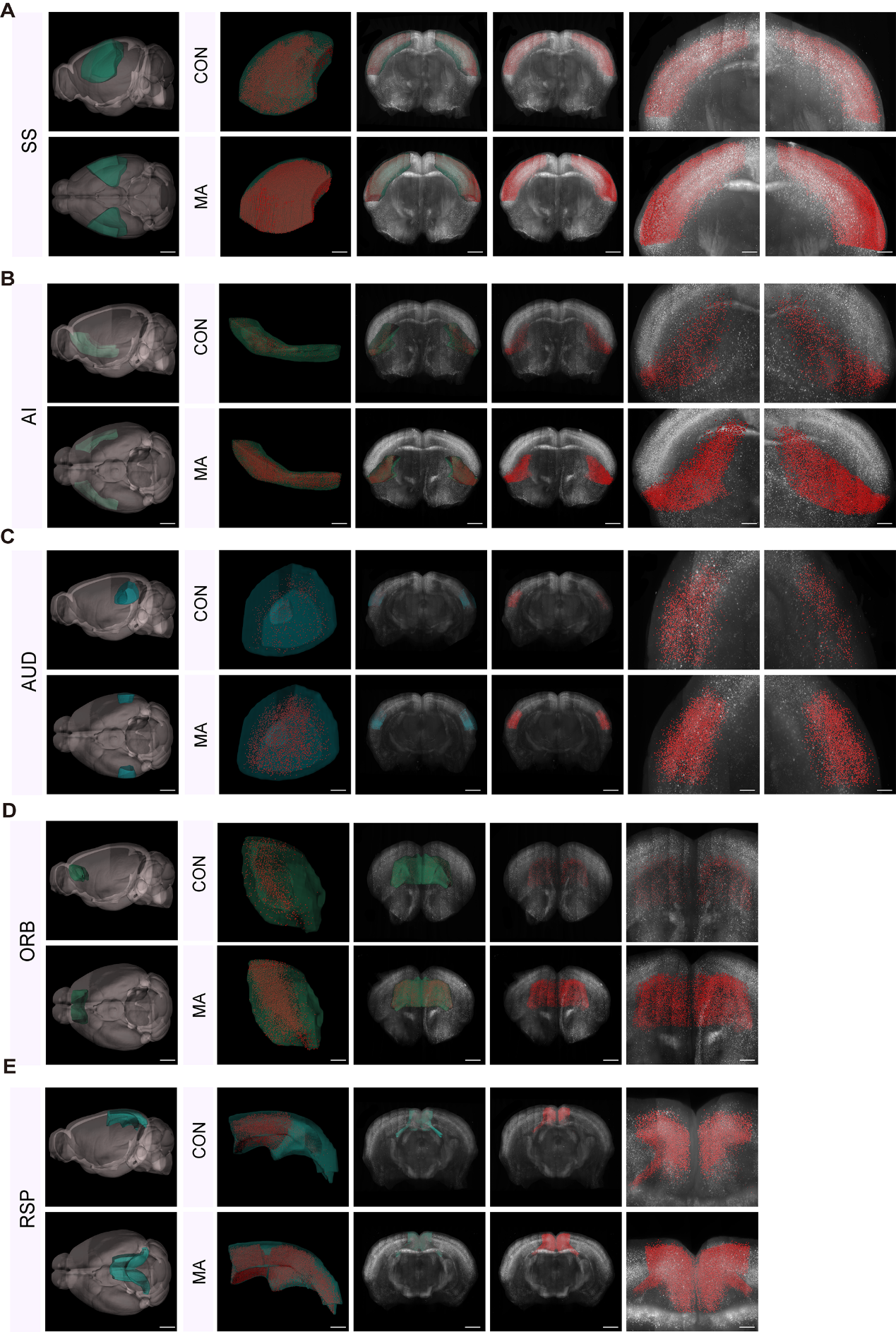


**Fig. S7 Three-dimensional reconstruction reveals increased FOS-EYFP^+^ neurons in multiple brain regions of MA mice compared to CON group**

A-D) Three-dimensional reconstruction across sagittal, coronal, and horizontal planes, showing significantly increased FOS-EYFP^+^ neuron counts in various regions (SS (A), AI (B), AUB (C), ORB (D), RSP (E)) of MA mice compared to controls. Scale bars: 1000 μm, 500 μm, 1000 μm, 100 μm and 500 μm (from left to right).


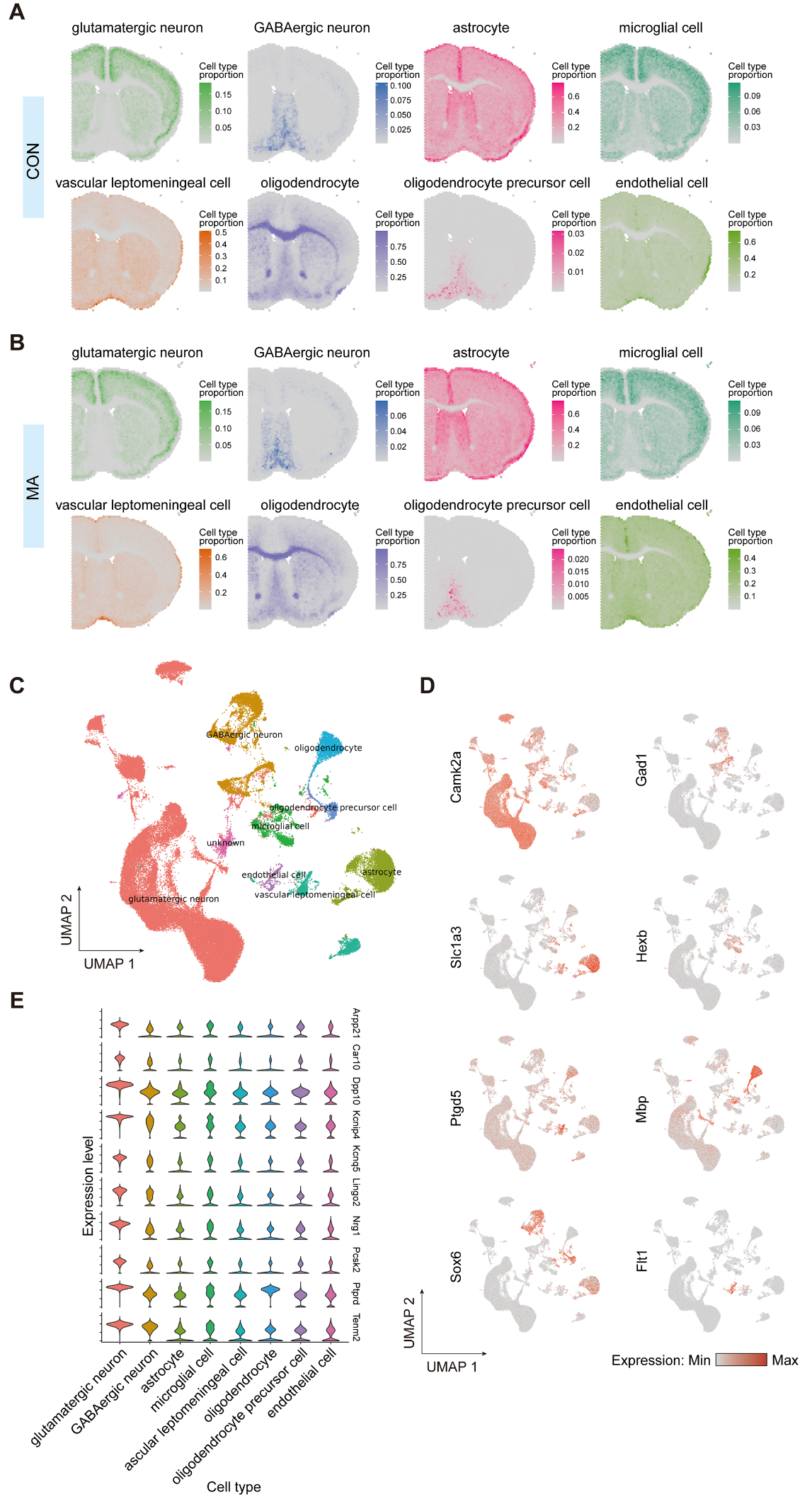


**Fig. S8 Spatial distribution, cell type identification, and marker gene expression profiles of cell populations in CON and MA groups**

A) Spatial mapping diagram of each cell population in the brain plane of the Con group. B) Spatial mapping diagram of each cell population in the brain plane of the MA group. C) Diagram for the identification of cell types by single-cell sequencing. D) Marker genes of each cell population. E) Specific expression of the TOP 10 genes in excitatory neurons.


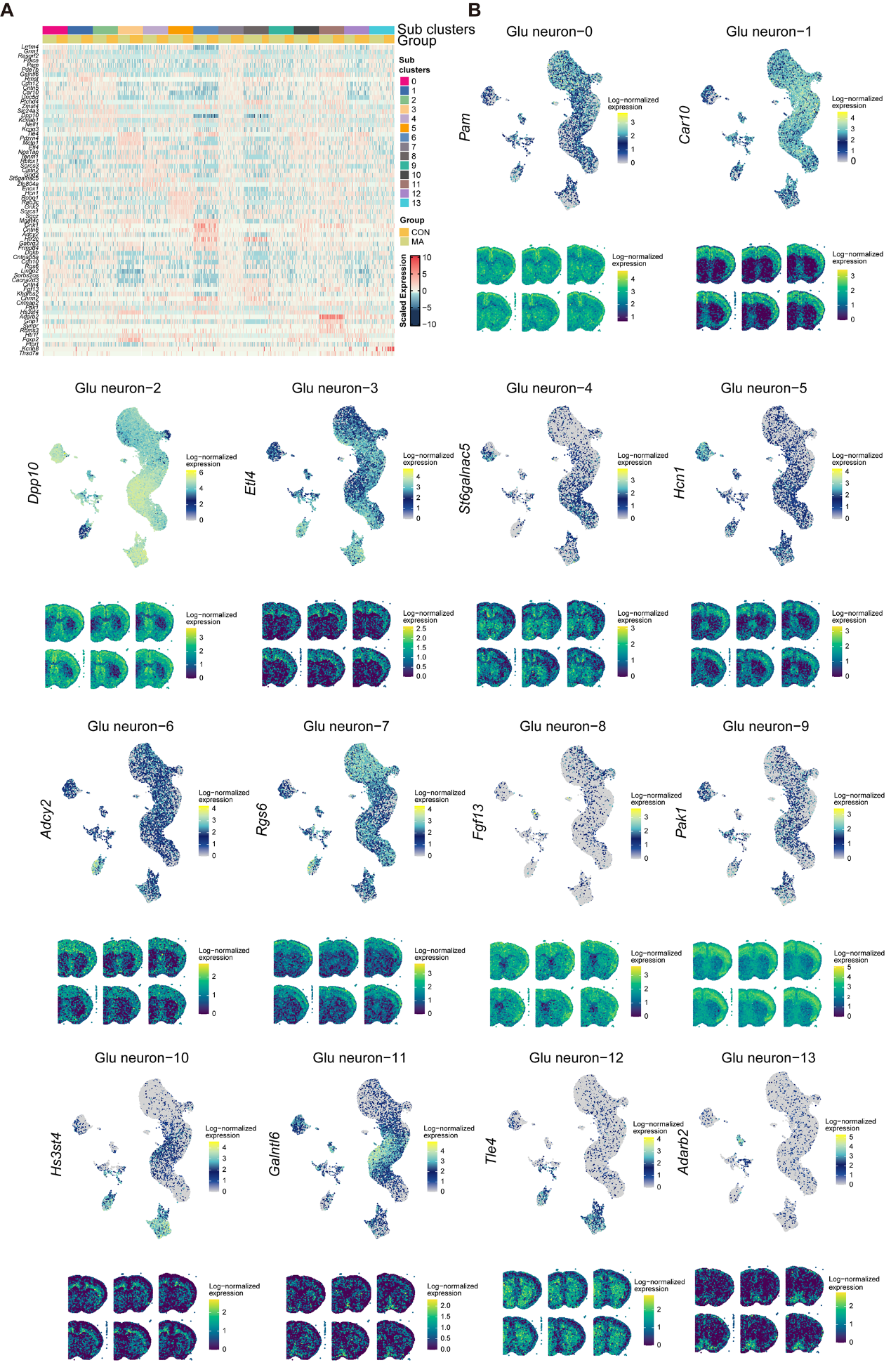


**Fig. S9 Characterization of glutamatergic neurons in ACC: heatmap of enriched marker genes and spatial distribution of neuronal subgroups**

A) Heatmap of enriched marker genes of glutamatergic neurons in 14 subpopulations of the ACC. B) Spatial distribution map of the marker genes for each neuronal subgroup.


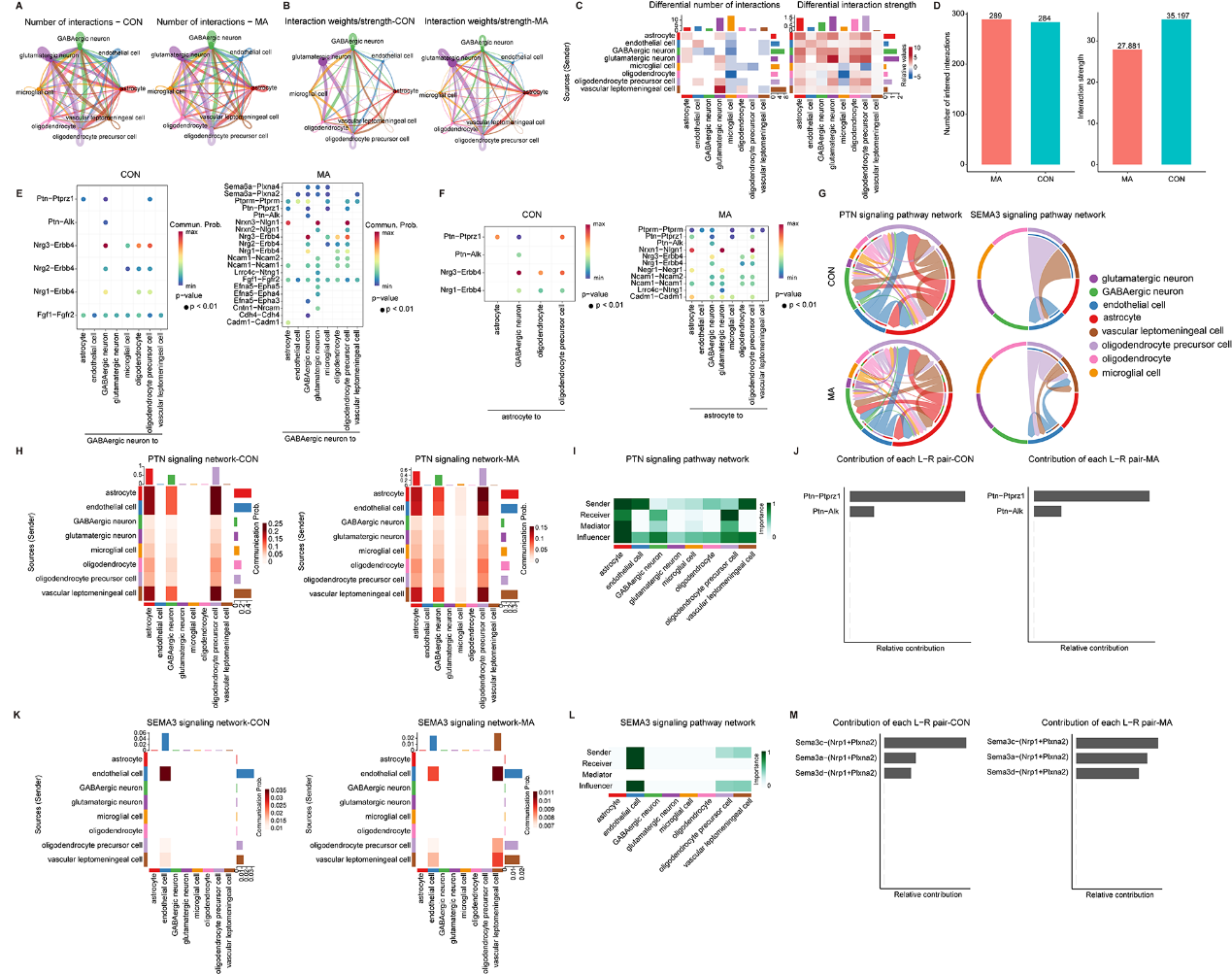


**Fig. S10 Comprehensive Analysis of cell-cell Interaction in** **CON and MA groups: from differential gene expression to signaling pathway networks**

A) Analysis of the number of differentially expressed genes related to cell interaction in single-cell sequencing of both the CON and MA groups. B) Analysis of the differential intensity of cell interaction in single-cell sequencing of both the CON and the MA group. C) Heatmap of the number and intensity of differentially expressed genes related to cell interaction. D) Histogram of the number and intensity of differentially expressed genes related to cell interaction. E) The expression differences of ligand-receptor pairs derived from GABAergic neurons between cells of the CON and MA groups. F) The expression differences of ligand-receptor pairs derived from astrocytes between cells of the CON and MA groups. G) The PTN (left) and SEMA3 (right) signaling pathway networks between cells of the CON and MA groups (with weak or without glutamatergic neurons, respectively. H) Heatmap of the PTN signals between cells of the CON and MA groups. I) The signaling pathway network of PTN between cells. J) Diagram of the contribution of ligand-receptor pairs in the PTN pathway between cells of the CON and MA groups. K) Heatmap of the PTN signals between cells of the CON and MA groups. L) The signaling pathway network of SEMA3 between cells. M) Diagram of the contribution of ligand-receptor pairs in the SEMA3 pathway between cells of the CON and MA groups.


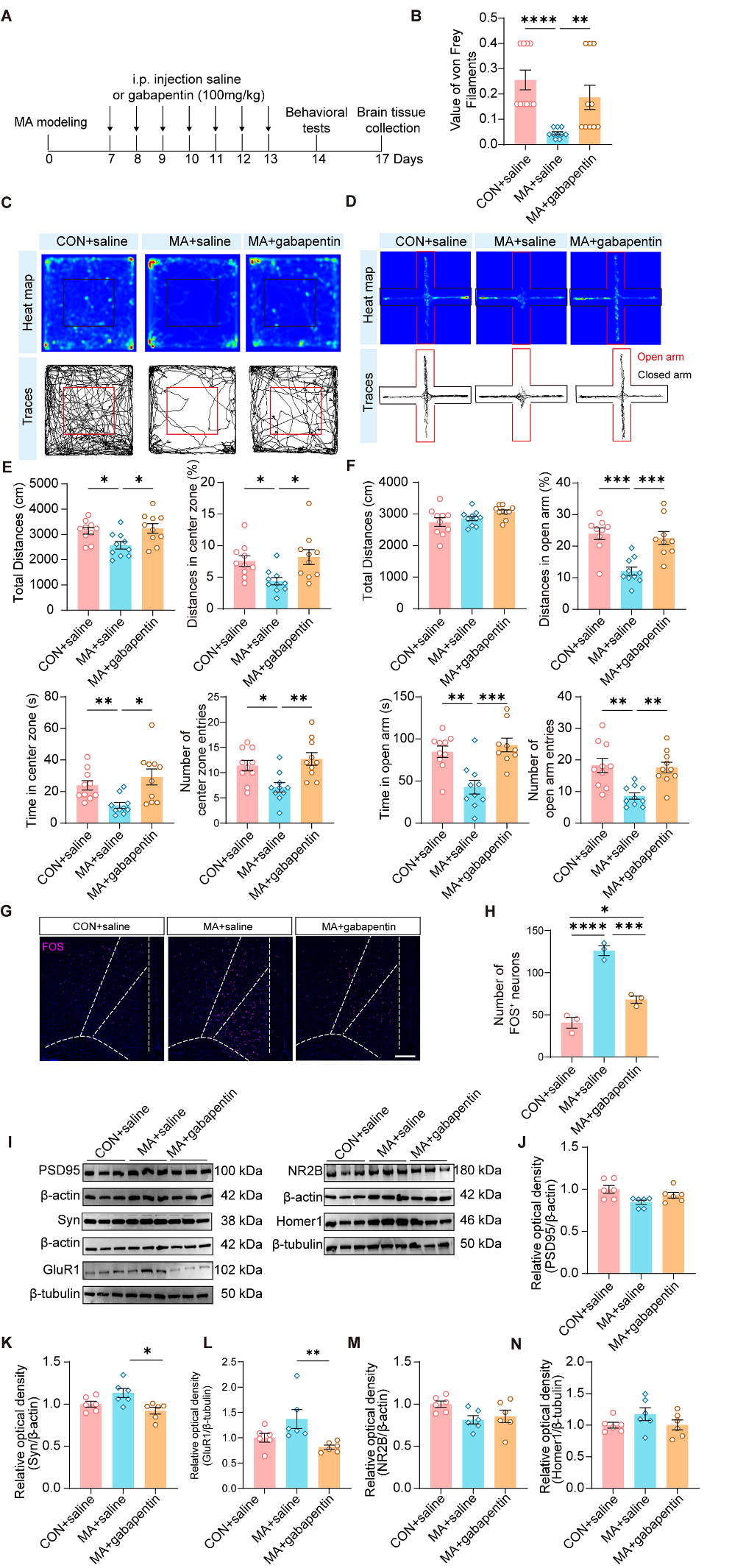


**Fig. S11 Gabapentin alleviates hyperalgesia and anxiety-like behaviors and reverses synaptic functional changes in MA mice**

A) A Experimental timeline: Mice underwent MA modeling, followed by intraperitoneal (i.p.) injections of saline or gabapentin (100 mg/kg), behavioral tests, and brain tissue collection. B) Statistical analysis of von Frey filament test (n = 10). C) Heat maps and traces in the open field test. D) Heat maps and traces in the elevated plus maze test. E) Quantification of total distances, distances in the center zone, time in the center zone, and number of center zone entries (n = 10). F) Quantification of total distances, distance in open arms, time in open arms, and number of open arm entries (n = 10). G) Representative images of FOS^+^ neuron staining in the ACC. Scale bars: 100 μm. H) Quantification of FOS^+^ neuron staining in the ACC (n = 3). I) Representative images of western blotting of PSD95, Syn, GluR1, NR2B, Homer1, and β-actin. J-N) Quantitative analyses of the relative optical densities of these proteins normalized to β-actin. MA + saline mice show altered optical densities of PSD95, Syn, GluR1, with NR2B and Homer1 unaffected; gabapentin normalizes these changes (n = 6). *P < 0.05, **P < 0.01, ***P < 0.001, ****P < 0.0001, by Welch ANOVA, One-way ANOVA, Kruskal-Wallis H test, *post hoc* Dunnet T multiple comparison, Tukey multiple comparison. Data are presented as mean ± SEM.


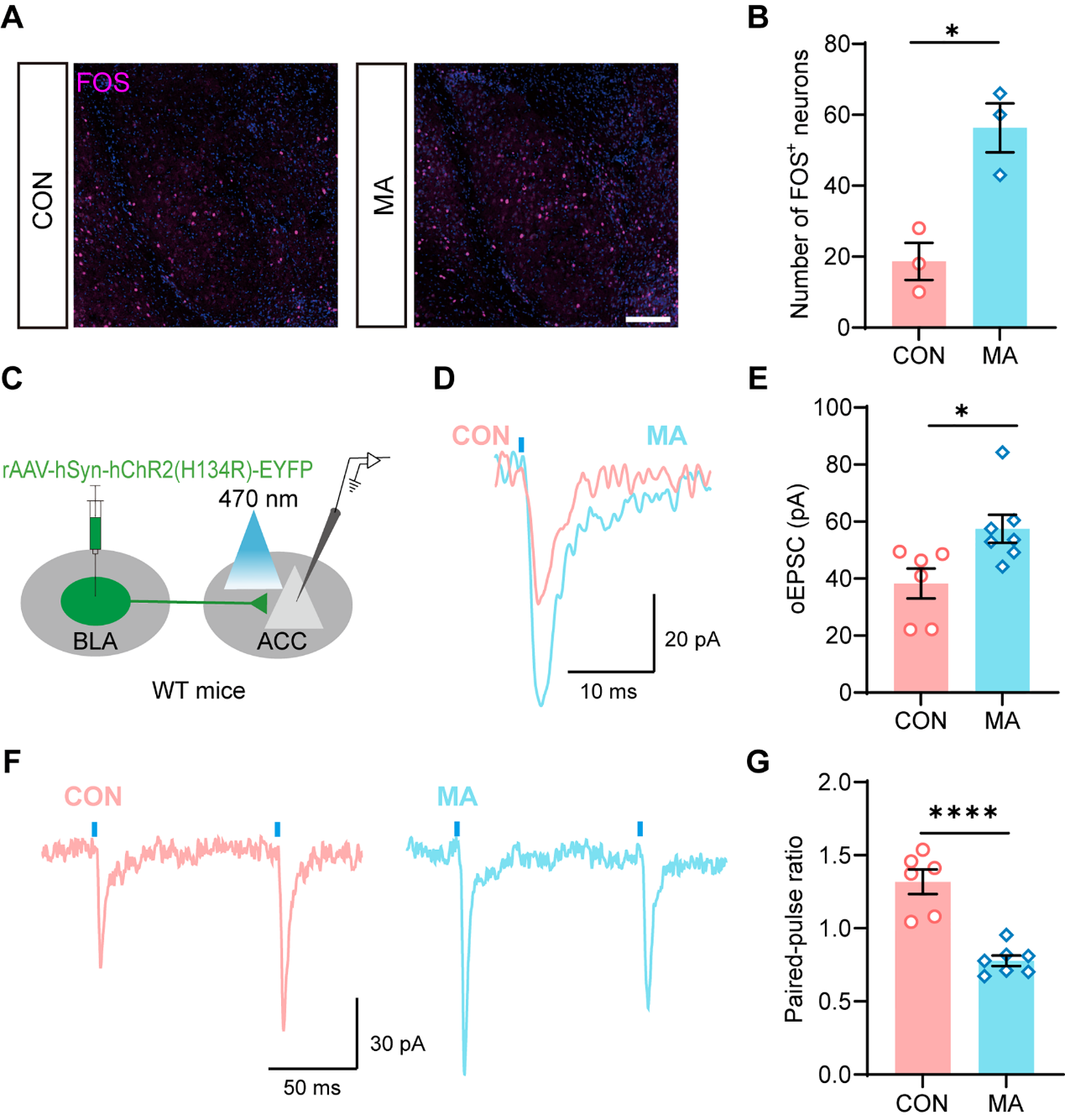


**Fig. S12 MA Model Alters Synaptic Plasticity of BLA-ACC Neurons**

A-B) FOS staining and quantification in the BLA, revealing significant activation in MA mice compared to CON (n = 3). Scale bars: 200 μm. C) Representative plot of the optogenetics-electrophysiology experimental setup. D) Representative plots of oEPSC in CON and MA model mice (n = 6 (CON), n = 7 (MA)). E) Statistical analysis of oEPSC (n = 6 (CON), n = 7 (MA)). (F) Representative plots of PPR in the CON and MA model mice. (G) Statistical analysis of PPR. (n = 6 (CON), n = 7 (MA)). *p < 0.05, ****p < 0.0001, by Mann-Whitney U test, Two-tailed unpaired t test. Data are presented as mean ± SEM.


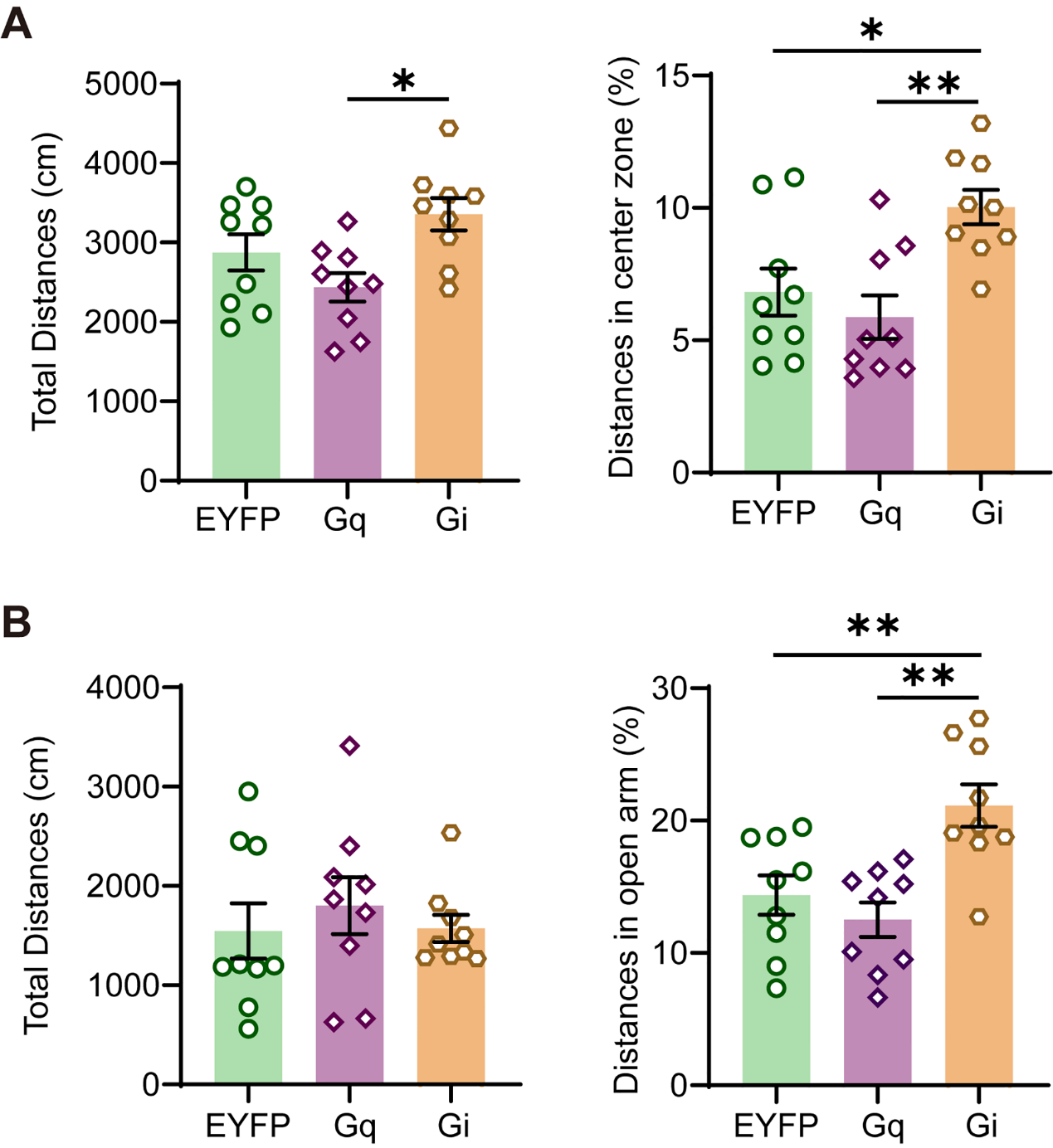


**Fig. S13 Chemogenetic modulation of changes in total distances in the male MA model mice.**

A) The left panel shows that the Gi group had significantly greater total distances traveled compared to Gq group. The right panel indicates the Gi group spent a significantly higher percentage of distance in the center zone compared to EYFP and Gq groups (n = 9). B) The left panel depicts total distances traveled with no indicated significance among groups. The right panel reveals that the Gi group spent a significantly higher percentage of distance in the open arm compared to the others (n = 9). * P < 0.05, **P < 0.01, by One-way ANOVA, Kruskal-Wallis H test, *Post hoc* Dunn multiple comparison, Tukey multiple comparison. Data are presented as mean ± SEM.


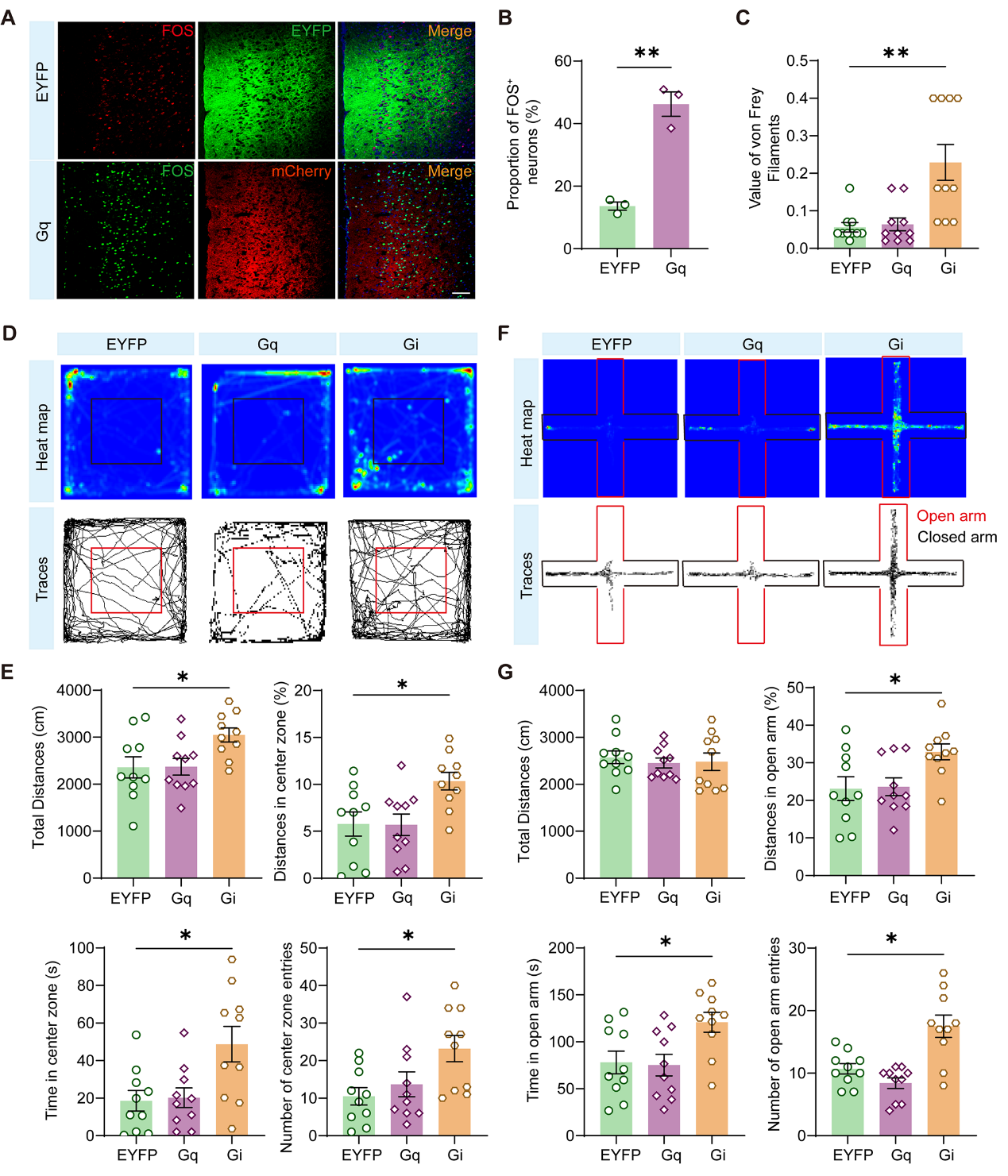


**Fig. S14 Chemogenetic regulation of hyperalgesia and anxiety-like behaviors in female MA mice**

A) Representative confocal images illustrating the colocalization of FOS with EYFP or mCherry (to label Gq-expressing neurons). Scale bars: 100 μm. B) Quantification of the proportion of FOS⁺ neurons among EYFP- or Gq-expressing populations. C) Analysis of von Frey filament-evoked pain responses. D) Heat maps (upper) visualize locomotor patterns, and traces (lower) depict animal trajectories. E) Quantification of total distances, distances in the center zone, time in the center zone, and number of center zone entries (n = 10). F) Heat maps (upper) and traces (lower) show movement in open and closed arms. G) Quantification of total distances, distance in open arms, time in open arms, and number of open arm entries (n = 10). *P < 0.05, **P < 0.01, by Two-tailed unpaired t test, Kruskal-Wallis H test, Welch ANOVA, One-way ANOVA, *post hoc* Dunn multiple comparison, Tukey multiple comparison, Dunnett T multiple comparison. Data are presented as mean ± SEM.
