# Supplementary material for: Synaptic Dysfunction in the Anterior Cingulate Cortex Underlies Pain‐Anxiety Comorbidity in a Mandibular Asymmetry Mouse Model
Source: Adv Sci (Weinh). 2025 Sep 24;12(46):e09509. doi: 10.1002/advs.202509509 (PMC12697876; doi:10.1002/advs.202509509)

Supporting Information

Synaptic Dysfunction in the Anterior Cingulate Cortex underlies Pain-Anxiety Comorbidity in a Mandibular Asymmetry Mouse Model

Zhaoyichun Zhang^1,2†^, Yanran Zhang^2†^, Jialin Si^3^, Honghui Mao^2^, Feng He^4^, Jin Ning^5^, Guaiguai Ma^2^, Xiaohua Chen^4^, Haoxiang Xiao^2^, Yuanyuan Zhu^2^, Haifeng Zhang^2^, Yifan Lu^2^, Qian Liu^2^, Meng Nian^3^, Shiquan Sun^5^, Shibin Yu^4^, Shengxi Wu^2^, Ze Fan^2,6*^, Zuolin Jin^1*^, Jing Huang^2*^

● Full Gels-1

● Full Gels-2

● Original images


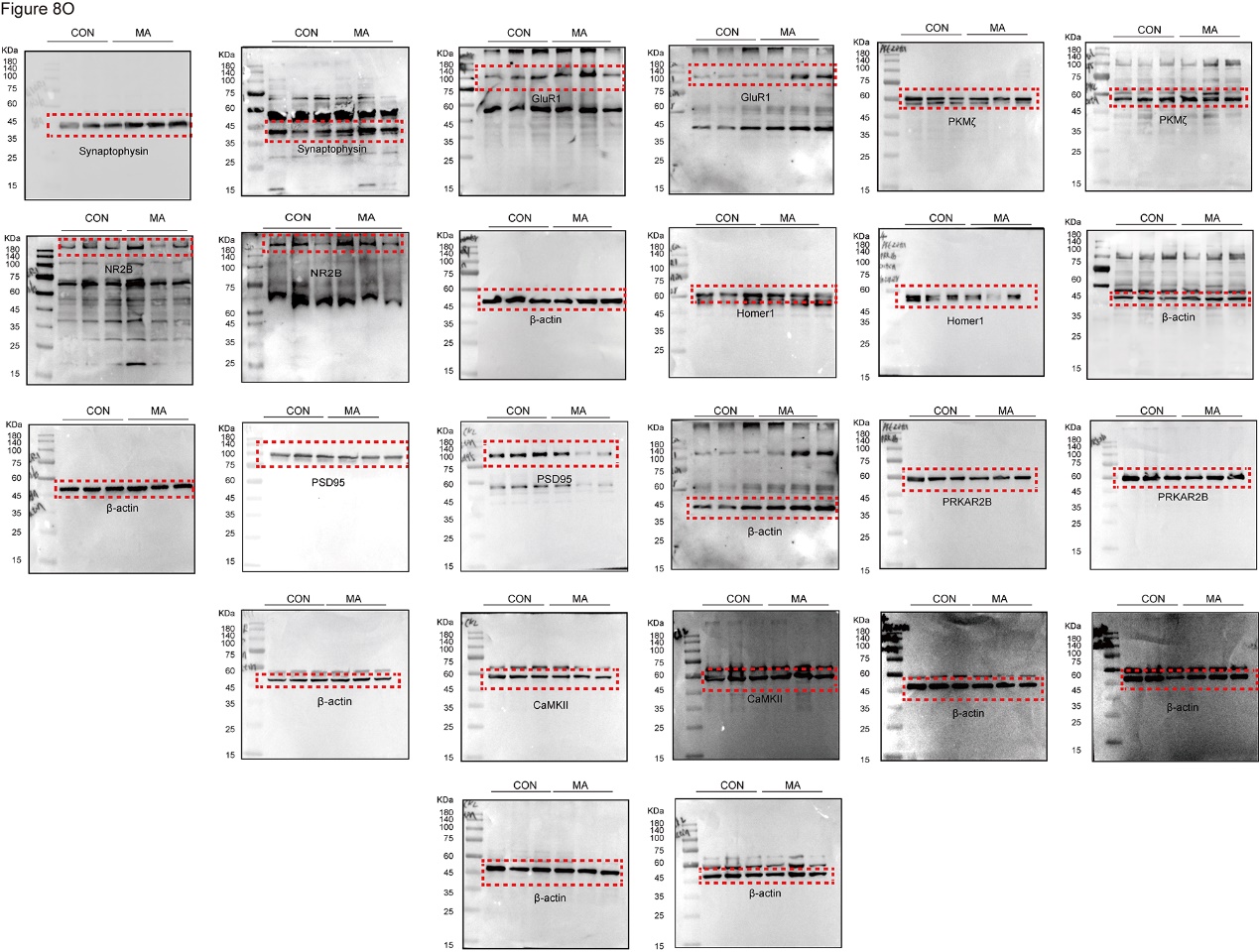


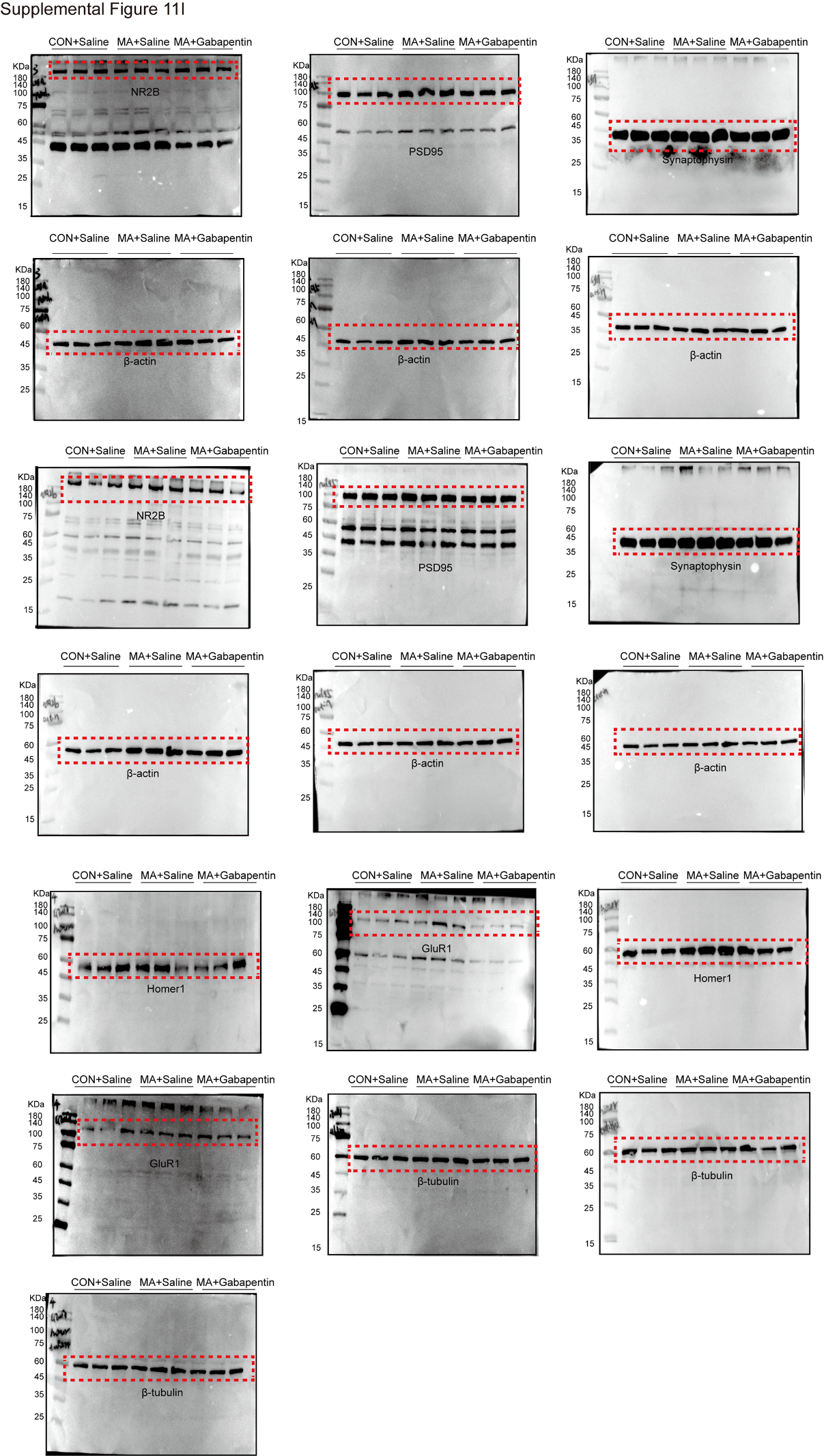


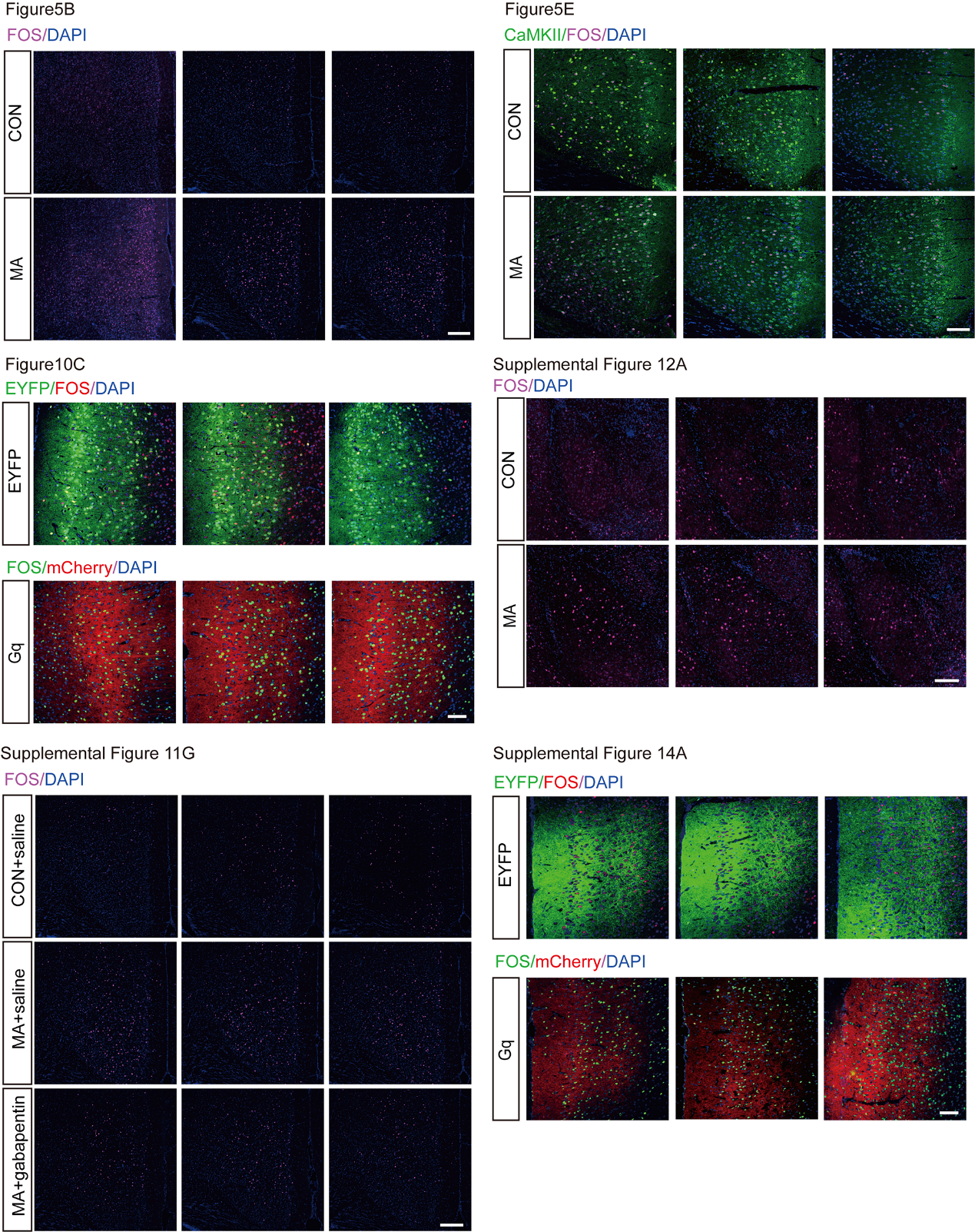

Supplement: Supplementary file 3 — Supporting Information [file ADVS-12-e09509-s002.docx]
